# Help-Seeking Self-Stigma and the Association Between Perceived Social Support and Psychological Distress Among Kuwait University Students: An Exploratory Cross-Sectional Moderation Analysis

**DOI:** 10.3390/ejihpe16090133

**Published:** 2026-09-02

**Authors:** Fatima Al-Ghadban, Ahmad Salman

**Affiliations:** Department of Public Health Practice, College of Public Health, Kuwait University, Kuwait City, Safat 13110, Kuwait; ahmad.salman@ku.edu.kw

**Keywords:** self-stigma, help-seeking, perceived social support, psychological distress, university students, Kuwait, moderation analysis, PHQ-4, SSOSH, confirmatory factor analysis

## Abstract

Background: Perceived social support is consistently associated with lower psychological distress among university students, but the strength of this association may vary. We examined whether help-seeking self-stigma modifies it. Methods: In a cross-sectional online survey, 507 Kuwait University students completed the Patient Health Questionnaire-4 (PHQ-4), the Multidimensional Scale of Perceived Social Support (MSPSS) and the Self-Stigma of Seeking Help Scale (SSOSH). Hierarchical linear regression tested the perceived support × self-stigma interaction, adjusting for age, sex, nationality and year of study; SSOSH structure was examined by confirmatory factor analysis. Results: Of 507 eligible students (80.9% female; mean age = 21.4 years, *SD* = 4.4), 458 had complete PHQ-4 data. The inverse association between perceived support and distress weakened as self-stigma increased (*B* = 0.003, 95% CI 0.001–0.005, *p* = 0.007; *p* = 0.040 with heteroscedasticity-consistent standard errors; Δ*R*^2^ = 0.015) and was not detectable at high self-stigma. The Arabic SSOSH was not unidimensional: positively and reverse-worded items formed uncorrelated factors (*r* = −0.01), and the interaction was carried by the reverse-worded items. Conclusions: Help-seeking self-stigma may index variation in this association, but the finding is exploratory and constrained by the measurement properties of the SSOSH in this population.

## 1. Introduction

Mental health concerns are common among university students, whose transition into higher education coincides with the typical age of onset for many mental health conditions while introducing substantial academic, social, and developmental challenges (Auerbach et al., 2018). Research across Arab populations identifies persistent stigma-related and cultural barriers to professional help-seeking (El-Shamy et al., 2023; Khatib et al., 2023), and evidence from Kuwait documents a substantial burden of depression, anxiety, and stress symptoms among university students (Alotaibi et al., 2024).

Perceived social support, the subjective perception that emotional, informational, and practical support is available from family, friends, and significant others, is among the most consistently identified protective factors associated with better mental health. Before introducing the literature, we state a framing qualification, because it defines what this study does and does not test. The stress-buffering model proposes that social support lessens the psychological impact of stressful experiences (S. Cohen & Wills, 1985), and it focuses on the interaction between stress exposure and support. No stressor-by-support interaction was examined here. The present question concerns something different: heterogeneity in the association between perceived support and distress according to a person-level characteristic. Our analyses should therefore be read as informed by the stress-buffering literature rather than as a test of it. Consistent with this perspective, systematic reviews of university students have shown that greater perceived social support is associated with lower psychological distress and better mental health (Ruihua et al., 2025), including among students living with mental illness (Vicary et al., 2025).

An important feature of perceived support is that it is conceptually and empirically distinct from support that is actually received. Meta-analytic evidence indicates that self-reported received and perceived support are only modestly correlated (Haber et al., 2007), and perceived availability is the more consistent predictor of mental health (Uchino, 2009). One account of why perceived support is beneficial is that it operates through everyday relational affect regulation rather than through crisis-time assistance (Lakey & Orehek, 2011); another is that support reduces distress through interpersonal emotion regulation, which requires the person to disclose difficulty to someone else (Marroquín, 2011). Both accounts imply that the benefit associated with perceived support is not automatic. It may depend on an individual’s willingness to acknowledge distress, disclose personal difficulties, and draw on the resources they believe are available. Consequently, psychological characteristics that discourage acknowledging psychological difficulty may influence the strength of this association.

Help-seeking self-stigma, defined as the internalization of negative beliefs about oneself for seeking psychological help, such as feeling inadequate or diminished for needing support, is a well-established barrier to accessing professional mental health services. Individuals with higher self-stigma may perceive seeking help as a sign of personal weakness, inadequacy, or failure, thereby reducing their willingness to seek support when experiencing psychological difficulties (Clement et al., 2015; Vogel et al., 2006). Self-stigma is conceptually distinct from public stigma, and meta-analytic evidence indicates that personal or internalized stigma, rather than perceived public stigma, is the component most consistently associated with reduced active help-seeking (Schnyder et al., 2017; Yu et al., 2023). Studies conducted in Arab and Gulf countries further suggest that concerns related to family reputation, anticipated social judgment, and feelings of shame contribute to help-seeking stigma, making stigma one of the most frequently reported barriers to mental health service utilization in the region (Al-Darmaki et al., 2016; Al-Krenawi et al., 2009; El-Shamy et al., 2023; Fekih-Romdhane et al., 2023b). Male university students have also been reported to endorse higher levels of mental health stigma than female students (Eisenberg et al., 2009; Nam et al., 2010), while studies conducted in Kuwait and neighboring countries suggest that recognition of psychological problems and perceived need for professional care may also differ by sex (Al-Krenawi et al., 2009).

Whether self-stigma about professional help extends to reluctance to use informal support from family and friends remains unclear. In a representative Belgian vignette study, anticipated self-stigma was associated with devaluing formal care but not significantly associated with attitudes toward informal help-seeking, whereas public stigma was associated with devaluing informal help (Pattyn et al., 2014). More recent work reports the opposite pattern, with internalized stigma predicting lower willingness to seek help from friends and family for personal and emotional problems (Kelly & Tonge, 2025). Related constructs point in the same direction: self-concealment, the tendency to actively withhold distressing personal information, is associated both with greater distress and with avoidance of psychological services (Larson et al., 2015; Cepeda-Benito & Short, 1998), and anticipated risks of self-disclosure predict help-seeking intentions (Vogel & Wester, 2003). Evidence from the United Arab Emirates indicates that self-stigma, loss of face, and self-disclosure jointly shape help-seeking attitudes among Gulf students (Heath et al., 2016). The question of whether professional help-seeking self-stigma indexes a broader reluctance to acknowledge or disclose distress therefore remains open, and the present study did not directly test it.

Although perceived social support and help-seeking self-stigma have each been widely studied as correlates of psychological distress and mental health service use, considerably less attention has been given to whether help-seeking self-stigma modifies the association between perceived social support and psychological distress. Meta-analytic work confirms that the support–distress association is systematically conditional on other variables (Rueger et al., 2016), and person-level characteristics such as attachment style (Dark-Freudeman et al., 2020) and the ability to receive compassion from others (Hermanto et al., 2016) have been shown to determine whether available support translates into lower distress. One recent study in Pacific Islander young adults reported that mental-health self-stigma moderated the association between social support and depression, anxiety, and stress (Sabado-Liwag et al., 2025). Evidence from university populations in Gulf countries remains particularly limited.

The rationale for expecting a scale about professional help to bear on informal support deserves to be stated precisely, because the two domains are conceptually distinct. The SSOSH measures negative self-evaluation attached specifically to seeking professional psychological help; the MSPSS measures perceived availability of support from family, friends, and a significant other. We do not propose that professional-help stigma acts directly on informal relationships. Rather, a person who evaluates themselves negatively for needing help may be less willing to acknowledge distress at all, and this reluctance is upstream of both domains. On that account, self-stigma is expected to mark students for whom perceived support is less likely to be converted into use, rather than to act upon support itself. This is a conjecture about a common antecedent, not a measured pathway, and the present study tests neither disclosure nor support use.

Examining this question in Kuwait is especially relevant given the cultural importance of family relationships, social reputation, and stigma surrounding mental health, all of which may shape students’ experiences of psychological distress and help-seeking. The present study therefore examined the associations among perceived social support, help-seeking self-stigma, and psychological distress in a cross-sectional sample of Kuwait University students.

Two main-effect hypotheses guided the analysis:

**H1.**  *Higher perceived social support would be associated with lower psychological distress. This follows from the large body of evidence linking perceived support to better mental health in student populations (Ruihua et al., 2025; Vicary et al., 2025) and from the stress-buffering and relational-regulation accounts of that association (S. Cohen & Wills, 1985; Lakey & Orehek, 2011).*

**H2.**  *Higher help-seeking self-stigma would be associated with higher psychological distress. This follows from evidence that internalized stigma is associated with reduced help-seeking and with the self-concealment of distress (Larson et al., 2015; Schnyder et al., 2017).*

A third question was addressed as an explicitly exploratory analysis. The interaction between perceived social support and help-seeking self-stigma emerged during exploratory examination of the data rather than being specified a priori; therefore, it is reported as an exploratory finding and interpreted cautiously throughout. The question was whether the inverse association between perceived social support and psychological distress would be weaker at higher levels of help-seeking self-stigma, as expected if self-stigma indexes a reluctance to acknowledge distress or mobilize available support. This study also reports a confirmatory factor analysis of the Arabic SSOSH, statistical diagnostics for common method variance, formal tests of the regression assumptions, and analyses of the PHQ-4 and MSPSS subscales.

## 2. Materials and Methods

### 2.1. Study Design and Setting

This cross-sectional study was conducted among students enrolled at Kuwait University. Data were collected between February and April 2026 via an anonymous, bilingual online questionnaire administered through Google Forms. Participants were recruited through convenience sampling using university-wide email announcements, student communication platforms, and college-specific networks to maximize participation across all academic colleges at Kuwait University.

Reporting follows the recommendations of Turk et al. (2018) for web-based survey studies, which combine the Survey Reporting Guideline (SURGE) and the Checklist for Reporting Results of Internet E-Surveys (CHERRIES); a completed checklist is provided as Table S1. The survey was open and voluntary rather than a closed survey administered to a defined list, and no registration or password was required. Because invitations were distributed through open channels, we did not capture the number of students who saw the invitation, so we cannot calculate view and participation rates as defined by Turk et al. (2018); we can report only the proportion of submitted questionnaires that were eligible and analyzable. The questionnaire comprised 11 screens, and no items were randomized. We used adaptive questioning only to route participants past sections that did not apply to them. Respondents could review and change their answers using the standard Google Forms back control before submission. No incentives were offered. No cookies, IP checks, or log-file analyses were used to detect multiple submissions from the same individual, which is acknowledged as a limitation. No personal identifiers were collected, so no additional data-protection mechanisms were required beyond the instrument’s anonymity.

The questionnaire displayed Arabic and English wording simultaneously for every item in the same online form; therefore, participants were not classified as completing separate Arabic- or English-language versions. Arabic wording for the PHQ-4, MSPSS, and BACE-30 was based on published Arabic versions that had undergone psychometric evaluation in Arabic-speaking samples (Alenezi et al., 2021; Fekih-Romdhane et al., 2023a; Obeid et al., 2024). We obtained the Arabic version of the Self-Stigma of Seeking Help Scale (SSOSH) from the scale developer’s official repository and attributed it to Fatima Rashed Al-Darmaki (Vogel, n.d.). However, we did not identify a peer-reviewed psychometric validation of the exact ten-item Arabic SSOSH wording used in the present Kuwait University sample. We did not undertake additional formal forward–backward translation or linguistic adaptation for this study, and we did not subject the bilingual questionnaire to a separate formal cognitive interview or pilot validation study before recruitment. Accordingly, the psychometric performance of the SSOSH was evaluated within the present sample using internal consistency, item-level analysis, and confirmatory factor analysis, and the absence of a formal validation of this Arabic version is acknowledged as a study limitation.

### 2.2. Participants

Eligible participants were students currently enrolled at Kuwait University who were aged 18 years or older and provided informed consent. At the beginning of the questionnaire, respondents were screened according to three eligibility criteria: age of at least 18 years, current enrollment at Kuwait University, and provision of informed consent.

A total of 543 questionnaires were submitted. Six respondents did not provide consent, 21 were not currently enrolled at Kuwait University, and nine were younger than 18 years, leaving 507 eligible participants.

The analytical dataset comprised all 507 eligible participants. The primary regression analysis included 458 participants with complete PHQ-4 data. The study size was determined by the number of eligible responses obtained during the recruitment period. No a priori power calculation was undertaken specifically for the exploratory interaction analysis; a post hoc consideration of the detectable effect size is given in Section 4.7. Participant characteristics are presented in Table 1. 

### 2.3. Measures

Psychological distress. Psychological distress was assessed with the Patient Health Questionnaire-4 (PHQ-4; Kroenke et al., 2009), an ultra-brief screening instrument consisting of two items assessing anxiety symptoms (Generalized Anxiety Disorder-2, GAD-2; example item: “Feeling nervous, anxious or on edge”) and two items assessing depressive symptoms (Patient Health Questionnaire-2, PHQ-2; example item: “Little interest or pleasure in doing things”). Participants rated how frequently they had been bothered by each symptom during the preceding two weeks using a four-point response scale ranging from 0 (“not at all”) to 3 (“nearly every day”). Total scores range from 0 to 12, with higher scores indicating greater psychological distress; subscale scores range from 0 to 6. Because the online response format inadvertently permitted more than one option to be selected for a PHQ-4 item, a valid score was calculated only when exactly one response option was recorded for each contributing item. Participants with an omitted or ambiguous multiple response on any of the four items were excluded from the complete-case primary analysis; subscale scores were computed whenever both contributing items were unambiguous, which is why the subscale analyses include more participants than the total-score analysis. Ambiguity was therefore handled at the level of the individual item rather than the scale: an item with more than one selected option was set to missing, items answered unambiguously by the same participant were retained, and it is these item-level missing values that were imputed in the analysis described in Section 2.5.6. Across the 507 eligible participants, 14, 17, 12, and 20 responses were ambiguous on the four items, respectively, and 49 participants had at least one such item; no eligible participant left any PHQ-4 item blank, so every exclusion arose from ambiguity rather than non-response. The GAD-2 and PHQ-2 subscales are analyzed separately in Section 3.9.

Perceived social support. Perceived social support was assessed using the 12-item Multidimensional Scale of Perceived Social Support (MSPSS; Zimet et al., 1988). The scale measures perceived support from three sources of four items each: significant others (example item: “There is a special person who is around when I am in need”), family (example item: “My family really tries to help me”), and friends (example item: “I can count on my friends when things go wrong”). Items are rated using the original seven-point Likert scale format ranging from 1 (“very strongly disagree”) to 7 (“very strongly agree”). Item scores were summed to generate a total score ranging from 12 to 84, with higher scores indicating greater perceived social support; subscale scores were computed as item means (range 1–7) following the developers’ scoring instructions. Subscale analyses are reported in Section 3.9.

Help-seeking self-stigma. Help-seeking self-stigma was assessed using the 10-item Self-Stigma of Seeking Help Scale (SSOSH; Vogel et al., 2006). The scale measures the extent to which seeking psychological help may negatively influence an individual’s self-perception and self-regard. Five items are positively worded with respect to stigma (example item: “I would feel inadequate if I went to a therapist for psychological help”) and five are reverse-worded (example item: “My self-esteem would increase if I talked to a therapist”) and are reverse-scored before summation. Items are rated from 1 (“strongly disagree”) to 5 (“strongly agree”). Scores are summed to produce a total score ranging from 10 to 50, with higher scores indicating greater help-seeking self-stigma. Previous cross-cultural research supports the scale’s applicability across diverse populations (Vogel et al., 2013). Because the psychometric performance of this Arabic wording had not previously been established, the internal structure of the scale was examined in the present sample by item analysis and confirmatory factor analysis before it was used as a moderator; the results of that examination are reported in Section 3.2 and materially qualify the interpretation of the findings.

Barriers to care. Barriers to accessing mental health care were assessed using the 30-item Barriers to Access to Care Evaluation (BACE-30; Clement et al., 2012; example item: “Concern that I might be seen as weak for having a mental health problem”). Each item assesses the extent to which a specific barrier has prevented, delayed, or discouraged the respondent from initiating or continuing professional mental health care. Items are rated from 0 (“not at all”) to 3 (“a lot”), producing a total score ranging from 0 to 90, with higher scores indicating greater perceived barriers.

### 2.4. Covariates

Four covariates were specified: age, sex, nationality, and year of study. Each was chosen based on prior evidence that it is associated with both perceived social support and psychological distress in student populations, so omitting it could confound the association of interest. Sex was included because female students consistently report higher levels of depressive and anxiety symptoms than male students (Auerbach et al., 2018; Salk et al., 2017) and because men and women differ in help-seeking attitudes and stigma endorsement (Nam et al., 2010; Pfeiffer & In-Albon, 2023). Age and year of study were included because psychological distress and perceived support vary across the course of university study and because the age of onset of common mental disorders falls within the typical undergraduate age range (Auerbach et al., 2018). Nationality was included because non-national students in Gulf settings may have smaller proximal family networks and different access to services (El-Shamy et al., 2023). Grade point average and college type were recorded but not included in the primary model because they are plausibly downstream of psychological distress, and including them would risk conditioning on a mediator. The parent survey also recorded perceived need for professional mental-health help and professional service use in the preceding 12 months. Neither was adjusted for, for the same reason: both are plausibly consequences of distress and of self-stigma rather than common causes of support and distress, so conditioning on them would block part of the association of interest and could induce collider bias. The estimand is accordingly the association between perceived support and psychological distress, and its variation across levels of self-stigma, adjusted only for demographic and educational characteristics that plausibly precede both. Residual confounding by unmeasured characteristics, including any operating through perceived need or care-seeking, cannot be excluded and is acknowledged in Section 4.7. One clarification follows, because the two roles are easily confused: perceived need for professional help is used as an auxiliary variable in the imputation model described in Section 2.5.6, where the aim is to predict missing values as well as possible and downstream variables are legitimate predictors, but it is not used as an adjustment covariate in any substantive model, where it would constitute overadjustment.

Nationality was dichotomized as Kuwaiti (coded 0) versus non-Kuwaiti (coded 1), the latter combining non-Kuwaiti students and those holding treated-as-Kuwaiti status, who together formed a smaller and administratively similar group. Year of study was dichotomized as first or second year (coded 0) versus third year or higher (coded 1) to preserve cell sizes. Sex was coded 0 for female and 1 for male. Age was analyzed as a continuous variable in years. Regression coefficients for these variables therefore express the difference in mean PHQ-4 score for the category coded 1 relative to the category coded 0.

### 2.5. Statistical Analysis

Analyses were conducted in IBM SPSS Statistics (version 31) and, for the confirmatory factor analyses, the common method variance diagnostics, the formal assumption tests, the delta-adjusted imputation, the inverse probability weighting and the generalized linear model, in Python 3.11 using NumPy and SciPy. This section describes, in order, the descriptive and psychometric analyses, the primary regression model, the procedures used to probe the interaction, the treatment of missing data, and the sensitivity analyses. All statistical tests were two-sided, and statistical significance was defined as *p* < 0.05. Continuous variables are reported to two decimal places and *p*-values to three decimal places, except where a smaller value is reported as *p* < 0.001.

#### 2.5.1. Descriptive and Psychometric Analyses

Descriptive statistics were used to summarize participant characteristics and scale scores. Internal consistency was assessed with Cronbach’s α and McDonald’s ω, the latter computed from a single-factor congeneric model. Item-level performance of the SSOSH was examined using item means, standard deviations, skewness, kurtosis, corrected item–total correlations, and α-if-item-deleted. Because no peer-reviewed validation of this Arabic SSOSH wording was available, we tested the scale’s internal structure using confirmatory factor analysis with maximum-likelihood estimation on the item correlation matrix. Model fit was evaluated with the model χ^2^, the comparative fit index (CFI), the Tucker–Lewis index (TLI), the root mean square error of approximation (RMSEA) with its 90% confidence interval, and the standardized root mean square residual (SRMR). Fit was judged against the combinational criteria proposed by Hu and Bentler (1999), namely CFI and TLI ≥ 0.95, RMSEA ≤ 0.06, and SRMR ≤ 0.08, while recognizing that these values are guidelines rather than absolute thresholds (Marsh et al., 2004). The same procedure was applied to the MSPSS to verify its three-factor structure.

#### 2.5.2. Assessment of Common Method Variance

Because all constructs were self-reported in a single administration, we assessed common method variance statistically and acknowledged it narratively. Harman’s single-factor test was conducted by submitting all 56 substantive items to an unrotated principal component analysis and examining the proportion of variance accounted for by the first component, and by fitting a single common factor to all items and evaluating its fit (Podsakoff & Organ, 1986). Because Harman’s test is widely recognized to be insensitive (Fuller et al., 2016; Podsakoff et al., 2003), it is interpreted alongside the analytical result that common method variance cannot generate spurious interaction effects and, if anything, attenuates them (Evans, 1985; Siemsen et al., 2010).

#### 2.5.3. Primary Regression Model

The primary analysis used hierarchical linear regression with the PHQ-4 total score as the dependent variable. Covariates (age, sex, nationality, and year of study) were entered in Block 1. The main effects of perceived social support and help-seeking self-stigma were entered in Block 2. The perceived social support × self-stigma interaction term was entered in Block 3. Perceived social support and help-seeking self-stigma scores were mean-centered before the creation of the interaction term to aid interpretation of the lower-order coefficients; centering does not alter the estimate, standard error, or significance of the interaction term itself (Dalal & Zickar, 2012; Echambadi & Hess, 2007). Centering constants were based on the full eligible sample (MSPSS mean 54.19; SSOSH mean 25.07). Effect size for the interaction is reported as Δ*R*^2^ and as Cohen’s *f*^2^ (J. Cohen, 1988).

#### 2.5.4. Planned Assumption Tests and Influence Diagnostics

We formally tested and reported all regression assumptions in Section 3.7 and Table S5. We assessed normality of residuals using the Shapiro–Wilk test, the Kolmogorov–Smirnov statistic, and standardized indices of skewness and kurtosis. We assessed homoscedasticity using the Koenker studentized Breusch–Pagan test, White’s general test, and the rank correlation between absolute residuals and fitted values. Functional form was assessed using Ramsey’s RESET test and by adding quadratic terms for each focal predictor. We assessed multicollinearity using variance inflation factors and tolerances. We assessed influence using standardized and studentized residuals, leverage values, Cook’s distance, and DFBETAS for the interaction coefficient. Screening thresholds were specified in advance: |standardized residual| > 3; leverage > 2*k*/*n*, the conventional twice-the-average-hat-value rule (Hoaglin & Welsch, 1978); Cook’s distance > 1 as the substantive criterion and >4/*n* as the more conservative screening criterion (Cook & Weisberg, 1982; Fox, 1991); and |DFBETAS| > 2/√*n* (Belsley et al., 1980). Because these thresholds flag cases for inspection rather than for automatic deletion, no case was excluded from the primary model; instead, the model was re-estimated with flagged cases removed and a leave-one-out analysis was conducted, both reported as sensitivity analyses.

#### 2.5.5. Probing the Interaction

We examined significant interaction effects using simple slope analyses estimating the association between perceived social support and psychological distress at low (−1 *SD*), mean, and high (+1 *SD*) levels of help-seeking self-stigma (Aiken & West, 1991). We generated conditional models by recentering the self-stigma variable at each level. Because fixed points are arbitrary, we also used the Johnson–Neyman technique to identify the region of the moderator over which the conditional association was statistically distinguishable from zero (Johnson & Fay, 1950; Preacher et al., 2006). Simple slopes were probed using the mean and standard deviation of SSOSH among the 458 complete-case participants (mean 24.87, *SD* 6.61).

#### 2.5.6. Missing Data

Because 49 participants (9.7%) had incomplete or ambiguous PHQ-4 item responses and were excluded from the primary regression analysis, we compared participants included in and excluded from the primary analysis on all available study variables. We compared categorical characteristics using chi-square tests and continuous variables using Welch’s *t*-tests, reporting test statistics, degrees of freedom, mean differences with 95% confidence intervals, and Cohen’s *d*. We then used three complementary approaches to the missing outcome. First, we imputed PHQ-4 items at the item level using fully conditional specification with categorical logistic models, generating 50 imputed datasets pooled by Rubin’s rules (Rubin, 1987; van Buuren, 2018). The imputation model included the remaining PHQ-4 items, age, sex, nationality, year of study, the centered MSPSS and SSOSH scores, their product term, the BACE-30 total score, and perceived need for professional mental-health help; PHQ-4 totals were reconstructed within each imputed dataset. Second, because imputation under the missing-at-random assumption cannot itself test that assumption, a delta-adjusted pattern-mixture sensitivity analysis was conducted in which a fixed offset was added to the imputed PHQ-4 scores of excluded participants, and the offset was varied to identify any tipping point at which the conclusion would change (Cro et al., 2020; Liublinska & Rubin, 2014). Third, inverse probability weighting was used as an alternative to imputation: a logistic model of the probability of having complete PHQ-4 data was fitted, and complete cases were weighted by the inverse of their estimated probability, with robust standard errors (Seaman & White, 2013).

#### 2.5.7. Exploratory Status and Sensitivity Analyses

The moderation analysis was not specified in a preregistered or dated analysis plan and was identified during exploratory data examination. At that stage, we evaluated only one candidate interaction term: the interaction between MSPSS total score and SSOSH total score. We selected it on conceptual grounds rather than after screening multiple candidate moderators by statistical significance, and we tested no other interaction term or omitted any from reporting. We constructed the nine-item SSOSH score after observing the primary interaction, prompted by the weak item–total performance of item 10, and used it solely as a sensitivity analysis.

Several further interaction terms are reported, and we state plainly that all of them were estimated after the original analysis rather than being specified in advance: the two wording-based SSOSH composites (Section 3.8), the three MSPSS subscales (Section 3.9), a quadratic self-stigma term (Section 3.7), and a three-way interaction with sex (Section 3.10). Every one of these is reported here, including those that were not statistically significant, so that no selective reporting arises from their addition. No multiplicity adjustment was applied to any of these tests. Accordingly, we report the primary moderation finding as exploratory and hypothesis-generating, and the additional interaction analyses as descriptive rather than confirmatory.

Several additional analyses evaluated the robustness of the interaction. The final model was re-estimated using HC3 heteroscedasticity-consistent standard errors, which are recommended by default in samples of this size (Long & Ervin, 2000; MacKinnon & White, 1985). We assessed the sensitivity of the interaction coefficient to individual observations by refitting the model 458 times, each time omitting one case. A bias-corrected and accelerated bootstrap with 5000 valid case-resampling replicates provided a confidence interval for the interaction (Efron, 1987). The interaction was re-estimated using a nine-item SSOSH score excluding item 10, using each wording-based SSOSH composite separately, with the BACE-30 total score included as a covariate, and using a Poisson generalized linear model with robust standard errors to accommodate the bounded, count-like distribution of the PHQ-4. All robustness analyses were conducted after identification of the interaction and are interpreted as supportive rather than confirmatory.

### 2.6. Ethical Approval

The study was conducted in accordance with the Declaration of Helsinki. Ethical approval was obtained from Kuwait University’s Health Sciences Center Ethical Committee on 3 November 2025 (Approval No. VDR/EC-2025-159). All participants provided informed consent prior to participation. The questionnaire was fully anonymous, and no personally identifiable information was collected.

## 3. Results

### 3.1. Descriptive Statistics and Internal Consistency

The primary regression analysis included 458 of the 507 eligible participants (90.3%) who had complete PHQ-4 data. Descriptive statistics and internal consistency estimates for all study measures are presented in Table 2. The mean PHQ-4 total score was 5.13 (*SD* = 3.24), and 191 participants (41.7%) scored at or above the conventional cut-point of 6, indicating at least moderate distress; 230 of 480 participants (47.9%) screened positive on the GAD-2 (score ≥ 3) and 200 of 478 (41.8%) on the PHQ-2. These figures are somewhat higher than general-population norms for the PHQ-4 (Löwe et al., 2010) and broadly consistent with reports from other university student samples (Khubchandani et al., 2016) and from the Gulf region (Al-Jayyousi et al., 2025; Alotaibi et al., 2024).

Internal-consistency estimates ranged from acceptable to excellent, except for the SSOSH. Cronbach’s α was 0.83 for the PHQ-4, 0.95 for the MSPSS (0.94, 0.92, and 0.94 for the significant-other, family, and friends subscales, respectively), 0.94 for the BACE-30, and 0.72 for the SSOSH. McDonald’s ω was 0.83, 0.95, and 0.94 for the PHQ-4, MSPSS, and BACE-30. For the SSOSH, ω estimated from a single-factor congeneric model was 0.61 for the ten-item score and 0.60 for the nine-item score; because that model fitted the data poorly (Section 3.2), ω is not an appropriate summary of this scale’s reliability in the present sample. Item-level analysis indicated that item 10 had a corrected item–total correlation of only 0.03 and that removing it raised α from 0.72 to 0.76. Table S2 reports full item-level statistics for the SSOSH.

### 3.2. Dimensionality of the Arabic SSOSH

Because the psychometric properties of this Arabic SSOSH wording had not previously been established, we examined the scale’s internal structure before interpreting it as a moderator. The results materially qualify the findings that follow and are therefore reported before the substantive analyses.

A unidimensional model of the ten scored items did not fit the data: χ^2^(35) = 931.93, *p* < 0.001, CFI = 0.508, TLI = 0.368, RMSEA = 0.225 (90% CI 0.213–0.238), SRMR = 0.215. Removing item 10 did not improve fit, χ^2^(27) = 848.78, *p* < 0.001, CFI = 0.502, TLI = 0.337, RMSEA = 0.245 (90% CI 0.231–0.260), SRMR = 0.223. Inspection of the loadings showed that the five reverse-worded items (2, 4, 5, 7, and 9) loaded close to zero on the general factor (standardized loadings 0.05–0.15 in absolute value), whereas the positively worded items loaded strongly (0.46–0.83). A two-factor solution corresponding exactly to item wording fitted well: χ^2^(26) = 83.88, *p* < 0.001, CFI = 0.968, TLI = 0.945, RMSEA = 0.066 (90% CI 0.051–0.082), SRMR = 0.027. Because the items are five-point ordinal scales, the analysis was repeated on the matrix of polychoric correlations, which does not assume interval-scaled response categories. The conclusion is unchanged: the mean polychoric correlation was 0.57 within the positively worded items and 0.54 within the reverse-worded items but 0.01 between the two sets, the unidimensional model again fitted poorly (CFI = 0.511, RMSEA = 0.266, SRMR = 0.245) with reverse-item loadings of 0.06 to 0.19 against 0.51 to 0.87 for the positively worded items, and the two-factor model again fitted far better (CFI = 0.942, SRMR = 0.032). Fit indices for all fitted models are given in Table S3, and the item correlation matrix is displayed in Figure S1.

Composites formed from the two item sets were each internally consistent (α = 0.82 and ω = 0.82 for both) but were statistically unrelated to one another, *r* = −0.01, *p* = 0.852. They also related to the other study variables in opposite directions. The positively worded composite was associated with higher psychological distress (*r* = 0.25, *p* < 0.001) and with greater perceived barriers to care (*r* = 0.40, *p* < 0.001) but not with perceived social support (*r* = −0.06, *p* = 0.200). The reverse-worded composite showed the opposite pattern, being associated with lower distress (*r* = −0.16, *p* < 0.001) and with lower perceived social support (*r* = −0.31, *p* < 0.001) but not with barriers to care (*r* = −0.05, *p* = 0.245). The SSOSH total score therefore sums two internally consistent but mutually unrelated components. By contrast, a three-factor model of the MSPSS corresponding to its published subscales fitted markedly better than a one-factor model, although its RMSEA remained above the conventional criterion (Table S3); the MSPSS was therefore scored as intended.

For transparency, and because the original submission used the ten-item total score, we retained that score as the primary measure throughout. The consequences of the two-factor structure for the substantive finding are reported in Section 3.8 and discussed in Section 4.2.

### 3.3. Common Method Variance

All 56 substantive items were submitted to an unrotated principal component analysis. The first component accounted for 22.7% of the total variance, and eleven components had eigenvalues greater than 1, together accounting for 67.3%. A single common factor fitted the 56 items very poorly, χ^2^(1484) = 11,859.2, *p* < 0.001, CFI = 0.356, TLI = 0.332, RMSEA = 0.124 (90% CI 0.122–0.126), SRMR = 0.164, and accounted for 21.4% of item variance. Restricting the analysis to the 26 items of the three focal scales, the first unrotated component accounted for 32.0% of variance. No single factor therefore dominated the item covariances. These results are reported in Table S4.

Harman’s test is a weak diagnostic test, and a null result does not establish the absence of method bias (Fuller et al., 2016; Podsakoff et al., 2003). Two further considerations are relevant. First, the focal finding of this study is an interaction, and both analytical and simulation work show that common method variance cannot create spurious interaction effects in moderated regression and instead attenuates true ones (Evans, 1985; Siemsen et al., 2010); an observed interaction is therefore conservative with respect to method bias. Second, the wording-based structure documented in Section 3.2 is itself a method effect, and it is addressed directly rather than through a global diagnostic.

### 3.4. Comparison of Included and Excluded Participants

Participants with complete and with incomplete or ambiguous PHQ-4 item responses did not differ significantly with respect to sex, χ^2^(1) = 0.28, *p* = 0.599; nationality, χ^2^(1) = 1.62, *p* = 0.203; year of study, χ^2^(1) = 0.02, *p* = 0.884; college type, χ^2^(1) = 2.45, *p* = 0.118; age, *t*(55.3) = −1.22, *p* = 0.226, *d* = −0.21; perceived social support, *t*(56.7) = 1.80, *p* = 0.078, *d* = 0.29; barriers to care, *t*(57.9) = −1.31, *p* = 0.195, *d* = −0.20; or grade point average, *t*(55.1) = 0.49, *p* = 0.628, *d* = 0.08. Excluded participants did report significantly higher help-seeking self-stigma than included participants (*M* = 26.96 versus 24.87; mean difference = −2.09, 95% CI −3.93 to −0.25; *t*(60.8) = −2.27, *p* = 0.027, *d* = −0.32). This difference was confined to the reverse-worded component (mean difference = −1.95, *t*(56.4) = −2.36, *p* = 0.022, *d* = −0.39); the positively worded component did not differ (mean difference = −0.14, *t*(56.1) = −0.19, *p* = 0.848, *d* = −0.03). Full comparisons are given in Table S6. Because missingness on the outcome was associated with the moderator, we report three complementary missing-data analyses in Section 3.10.

### 3.5. Bivariate Associations

Table 3 presents zero-order Pearson correlations among the study variables. Psychological distress was inversely correlated with perceived social support (*r* = −0.16, *p* < 0.001), consistent with H1, and positively correlated with perceived barriers to care (*r* = 0.27, *p* < 0.001). Contrary to H2, help-seeking self-stigma was not significantly correlated with psychological distress (*r* = 0.05, *p* = 0.336). Higher self-stigma was associated with lower perceived social support (*r* = −0.28, *p* < 0.001) and greater perceived barriers to care (*r* = 0.22, *p* < 0.001). Perceived social support was not significantly associated with barriers to care (*r* = −0.05, *p* = 0.280). Among the MSPSS subscales, family support showed the strongest inverse association with distress (*r* = −0.19, *p* < 0.001), followed by support from a significant other (*r* = −0.13, *p* = 0.004) and from friends (*r* = −0.10, *p* = 0.025).

### 3.6. Primary Moderation Model

**Figure 1:**
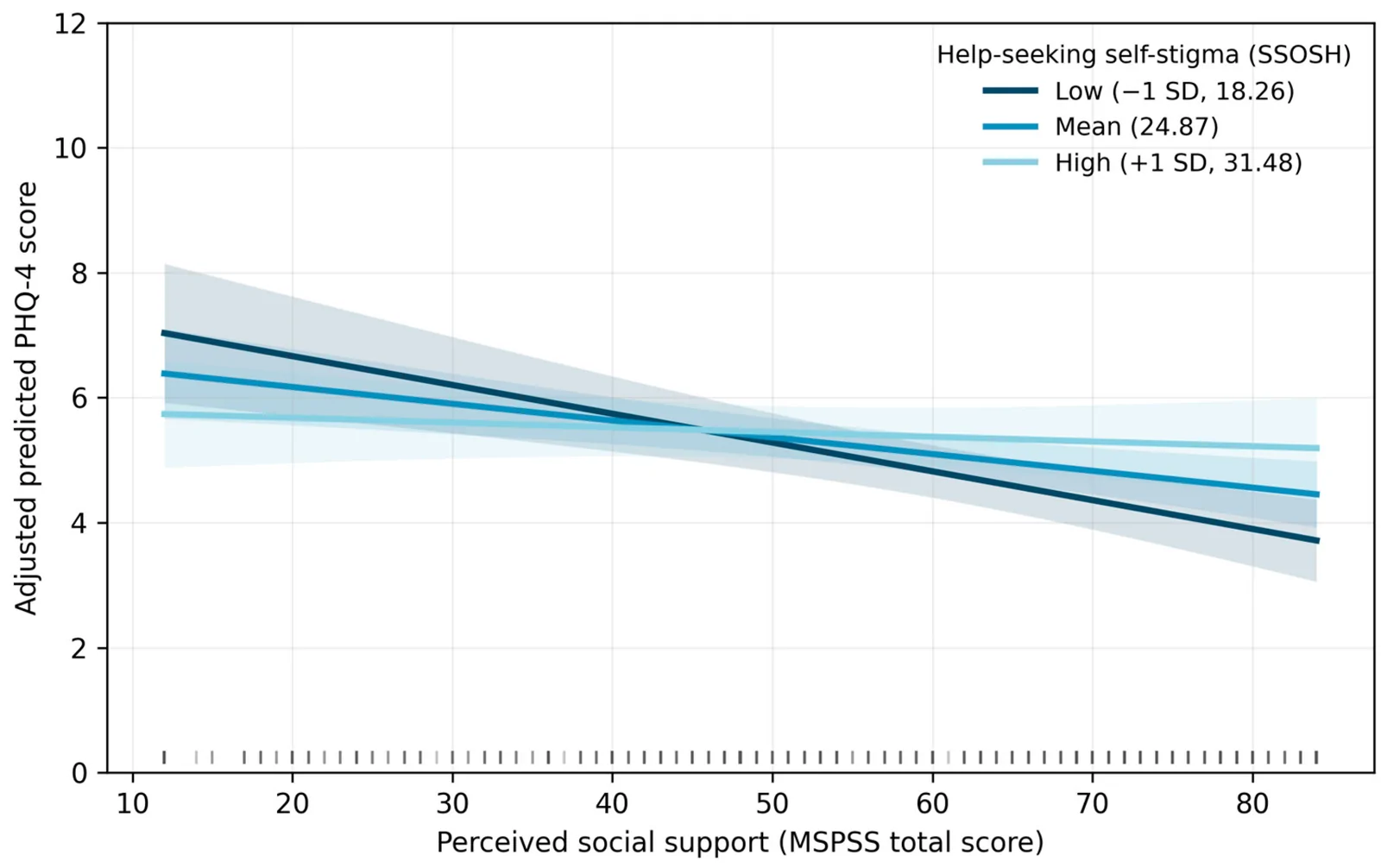
Adjusted predicted PHQ-4 scores across perceived social support according to help-seeking self-stigma. Predictions were generated from the adjusted linear regression model and averaged over the observed distributions of age, sex, nationality, and year of study. SSOSH levels correspond to the complete-case mean of 24.87 and one standard deviation below and above the mean, corresponding to scores of 18.26 and 31.48. Shaded ribbons show 95% confidence intervals, and tick marks along the horizontal axis show observed MSPSS values. The vertical axis displays the full PHQ-4 range of 0–12; the same predictions on an expanded vertical axis are shown in Figure S2. The interaction was exploratory and crossover in form.

Hierarchical linear regression examined whether help-seeking self-stigma modified the association between perceived social support and psychological distress after adjustment for age, sex, nationality, and year of study (Table 4; Figure 1).

The final model was statistically significant, *F*(7, 450) = 5.04, *p* < 0.001, explaining 7.3% of the variance in PHQ-4 scores (*R*^2^ = 0.073; adjusted *R*^2^ = 0.058). Inclusion of the interaction significantly improved model fit, Δ*R*^2^ = 0.015, *F*(1, 450) = 7.27, *p* = 0.007, corresponding to Cohen’s *f*^2^ = 0.016.

The MSPSS × SSOSH interaction was statistically significant (*B* = 0.003, 95% CI 0.001–0.005, β = 0.12, *t* = 2.70, *p* = 0.007), indicating that higher levels of self-stigma were associated with a weaker inverse association between perceived social support and psychological distress. At the mean level of help-seeking self-stigma, higher perceived social support remained significantly associated with lower psychological distress (*B* = −0.027, 95% CI −0.042 to −0.012, *p* < 0.001). Of the covariates, only sex was independently associated with psychological distress: male students had lower mean PHQ-4 scores than female students after adjustment (*B* = −1.312, 95% CI −2.081 to −0.543, *p* < 0.001). Age, nationality, year of study, and the main effect of help-seeking self-stigma were not statistically significant. Multicollinearity was not evident (all variance inflation factors ≤ 1.24; all tolerances ≥ 0.81).

### 3.7. Regression Assumptions and Influence Diagnostics

Assumption tests are summarized here and reported in full in Table S5. Residuals departed modestly from normality (Shapiro–Wilk *W* = 0.984, *p* < 0.001; skewness = 0.31, *SE* = 0.11; kurtosis = −0.48, *SE* = 0.23), a deviation of a magnitude that is not consequential for coefficient estimation at this sample size. The assumption of constant error variance was not satisfied: the Koenker studentized Breusch–Pagan test was significant, *LM* = 24.32, *df* = 7, *p* = 0.001, as was White’s general test, *LM* = 78.81, *df* = 31, *p* < 0.001, and absolute residuals correlated weakly with fitted values (Spearman ρ = 0.14, *p* = 0.004). We note for transparency that the simpler single-degree-of-freedom check of regressing the squared standardized residual on the standardized predicted value is not significant (*F*(1, 456) = 2.52, *p* = 0.113): error variance in these data varies systematically with the predictors but not monotonically with the fitted value, which is why the omnibus tests detect it, and the single-predictor test does not. Heteroscedasticity is expected for a bounded screening score and is why HC3 standard errors are reported alongside conventional standard errors throughout; the HC3 results in Section 3.10 should be regarded as the more trustworthy inference. Functional form was adequate overall (Ramsey RESET *F*(2, 448) = 1.78, *p* = 0.170), and adding a quadratic term for perceived social support was not informative (*B* = −0.00002, *p* = 0.965); a quadratic term for self-stigma was statistically significant (*B* = 0.008, *p* = 0.002) and did not attenuate the interaction, which remained significant when it was included (*B* = 0.004, *p* = 0.001). The Durbin–Watson statistic was 1.86.

No case had a standardized or studentized residual exceeding 3 in absolute value (range −1.99 to 2.52). The largest Cook’s distance was 0.078, far below the substantive threshold of 1; 24 cases (5.2%) exceeded the conservative 4/*n* screening threshold of 0.009. Maximum leverage was 0.107, with 33 cases exceeding 2*k*/*n* = 0.035, where *k* is the number of estimated parameters including the intercept (*k* = 8); leverage is reported as the uncentered hat value, and the corresponding centered leverage, which some software reports by default, is 0.105. Re-estimating the model after excluding all cases flagged by the 4/*n* rule did not weaken the interaction; it strengthened it (*B* = 0.005, *SE* = 0.001, *t* = 4.50, *p* < 0.001, *n* = 434). Together with the leave-one-out analysis reported in Section 3.10, this indicates that influential observations do not produce the interaction.

### 3.8. Wording-Based Decomposition of the Moderator

**Figure 2:**
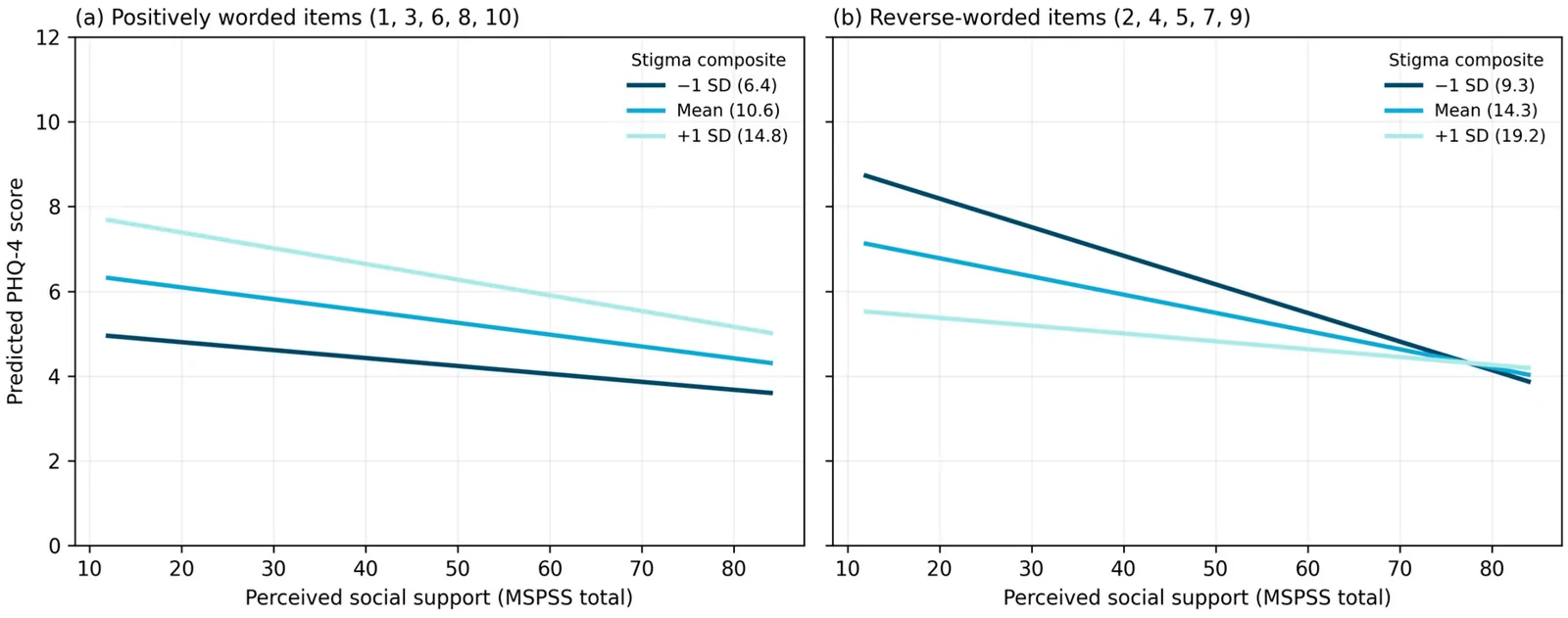
Adjusted predicted PHQ-4 scores across perceived social support at three levels of each wording-based SSOSH composite. Panel (**a**) uses the five positively worded items; panel (**b**) uses the five reverse-worded items. Predictions are adjusted for age, sex, nationality, and year of study, evaluated at their sample means. The convergence of the three lines in panel (**b**) and their near-parallel form in panel (**a**) show that the moderation reported in Section 3.6 is attributable to the reverse-worded items.

Because the SSOSH total score comprises two unrelated components (Section 3.2), we re-estimated the moderation with each component in turn (Table 5; Figure 2). The reverse-worded items carried the interaction entirely. Using the reverse-worded composite as the moderator, the interaction was larger than with the total score (*B* = 0.005, *SE* = 0.001, *t* = 4.00, *p* < 0.001, Δ*R*^2^ = 0.031). Using the positively worded composite, no interaction was detected, and the point estimate was in the opposite direction (*B* = −0.002, *SE* = 0.002, *t* = −1.35, *p* = 0.177, Δ*R*^2^ = 0.004); excluding item 10 from that composite did not change this (*B* = −0.001, *p* = 0.550). When both composites and both of their interactions with perceived social support were entered simultaneously, only the reverse-worded interaction remained significant (*B* = 0.003, *p* = 0.006), while the positively worded interaction did not (*B* = −0.001, *p* = 0.607).

The two composites also behaved differently as main effects in that combined model. Higher scores on the positively worded composite were associated with higher distress (*B* = 0.212, *p* < 0.001) and higher scores on the reverse-worded composite with lower distress (*B* = −0.114, *p* < 0.001), and the model explained substantially more variance than the total-score model (*R*^2^ = 0.192 versus 0.073). Section 4.2 develops the substantive implications.

### 3.9. Probing the Interaction and Subscale Analyses

Simple slope analyses were conducted at low (−1 *SD*), mean, and high (+1 *SD*) levels of help-seeking self-stigma (Table 6; Figure 1). Among students reporting low self-stigma, higher perceived social support was associated with lower psychological distress (*B* = −0.046, *p* < 0.001). This inverse association was weaker at the mean level of self-stigma (*B* = −0.027, *p* < 0.001), and at high self-stigma the estimated slope was close to zero and not statistically distinguishable from zero (*B* = −0.008, 95% CI −0.027 to 0.012, *p* = 0.454). The confidence interval at high self-stigma is compatible with a small inverse association, with no association, or with a very small positive association; therefore, the correct statement is that the association is no longer statistically detectable at high self-stigma, not that support has been shown to lose its protective value.

The interaction was crossover in form: at lower MSPSS scores, predicted PHQ-4 scores were not higher among students with higher SSOSH scores, and the ordering of the predicted values reversed as MSPSS increased. Accordingly, the interaction should not be interpreted as evidence that self-stigma is uniformly associated with higher distress across the observed range of social support.

A Johnson–Neyman analysis identified the value of self-stigma above which the conditional association was no longer statistically distinguishable from zero. Using conventional standard errors, this boundary was an SSOSH score of 28.43, below which 64.2% of complete cases fell; using HC3 standard errors, it was 27.54, below which 58.5% fell. Because the sample mean was 24.87 and the boundary lies well within one standard deviation of it, the region in which the association is statistically detectable covers only around three-fifths of the sample. This is a real limitation on the practical reach of the finding and is discussed in Section 4.7. The boundary is an inferential property of this model and this sample size, not a clinical threshold.

Because a regression coefficient of 0.003 per MSPSS point is difficult to interpret substantively, Table 7 expresses the same model as predicted differences in PHQ-4 score across realistic portions of the observed distribution of perceived social support. Moving from the 10th to the 90th percentile of perceived support, a shift of 56 scale points corresponds to a predicted reduction of 2.58 PHQ-4 points among students at low self-stigma, 1.50 points at the mean, and 0.42 points at high self-stigma. Against an observed PHQ-4 standard deviation of 3.24, these correspond to roughly 0.80, 0.46, and 0.13 standard deviations. Across the interquartile range, the corresponding reductions are 1.38, 0.80, and 0.23 points. The difference between the low- and high-stigma conditions is therefore material at the extremes of the support distribution and negligible in the middle.

The crossover form of the interaction can be expressed in the same units, which shows why a directional reading is not warranted. Comparing students one standard deviation above and one standard deviation below the mean on self-stigma, the predicted difference in PHQ-4 score is −0.83 points at low perceived support (10th percentile; 95% CI −1.91 to 0.25, *p* = 0.130), 0.38 points at median support (95% CI −0.25 to 1.01, *p* = 0.236), and 1.32 points at high perceived support (90th percentile; 95% CI 0.39 to 2.26, *p* = 0.006). The only region in which self-stigma level is associated with a statistically detectable difference in distress is therefore at high perceived support, and the point estimate reverses sign at low support. This is the quantitative expression of the crossover noted above, and it is why the finding is described throughout as heterogeneity in an association rather than self-stigma diminishing a protective effect.

Whether the anxiety and depression components of the PHQ-4 behave differently, and whether the MSPSS subscales do, was examined next. Both sets of analyses are reported in Table 8. The interaction was evident for the GAD-2 anxiety subscale (*B* = 0.0019, *p* = 0.002, HC3 *p* = 0.010, Δ*R*^2^ = 0.020) and weaker for the PHQ-2 depression subscale (*B* = 0.0012, *p* = 0.042), where it did not survive HC3 estimation (*p* = 0.127). The depression result is fragile in a further respect that we report explicitly: restricting the model to the 458 participants with complete data on all four PHQ-4 items, rather than the 478 with both PHQ-2 items, renders it non-significant (*B* = 0.0011, *p* = 0.068; HC3 *p* = 0.169). The anxiety result is not sensitive to this choice (*n* = 458: *B* = 0.0018, *p* = 0.003; HC3 *p* = 0.012). We therefore regard the interaction as supported for anxiety symptoms and not established for depressive symptoms. Conversely, the main effect of perceived social support was clearer for depression (*B* = −0.018, *p* < 0.001) than for anxiety (*B* = −0.007, *p* = 0.128). Among the MSPSS subscales, the interaction was strongest for family support (*B* = 0.033, *p* = 0.007, HC3 *p* = 0.021), weaker for friends (*B* = 0.022, *p* = 0.043, HC3 *p* = 0.093), and not significant for support from a significant other (*B* = 0.021, *p* = 0.062). When all three subscales were entered simultaneously, no individual subscale interaction remained significant, which is unsurprising given intercorrelations of 0.64 to 0.72 among them; the subscales should therefore not be treated as independently informative.

### 3.10. Robustness and Missing-Data Analyses

We evaluated the interaction across twelve further analyses, summarized in Table 9. It remained statistically significant under HC3 heteroscedasticity-consistent standard errors, although with reduced precision (*B* = 0.0029, HC3 *SE* = 0.0014, *t*(450) = 2.06, *p* = 0.040, 95% CI 0.0001–0.0057). Because the homoscedasticity assumption was formally violated (Section 3.7), this is the appropriate primary inference, and the finding should be described as significant at *p* = 0.040 rather than *p* = 0.007. We applied HC3 estimation to every model in Table 9 for which it was applicable, and the interaction remained statistically significant under robust estimation in every case except the model using the positively worded composite, which was not significant under either estimator. A bias-corrected and accelerated bootstrap with 5000 resamples produced a 95% confidence interval of 0.0002–0.0056, excluding zero. A leave-one-out analysis re-estimating the model 458 times produced coefficients ranging from 0.0027 to 0.0036, positive in every model. Adjustment for barriers to care did not alter the interaction (*B* = 0.0027, *p* = 0.010; HC3 *p* = 0.036) while nearly doubling the explained variance (*R*^2^ = 0.139); barriers to care were independently associated with distress (*B* = 0.047, *p* < 0.001). A Poisson generalized linear model with robust standard errors, fitted to accommodate the bounded, count-like outcome, also supported the interaction (*B* = 0.0006, *z* = 2.29, *p* = 0.022).

Three analyses addressed the differential missingness on the outcome. Item-level multiple imputation of the PHQ-4 by fully conditional specification (50 datasets, pooled by Rubin’s rules) yielded a pooled interaction of *B* = 0.0032, *SE* = 0.0011, *t* = 2.96, *p* = 0.003. A delta-adjusted pattern-mixture analysis was then conducted using a re-specified imputation of the PHQ-4 total score, which, under the missing-at-random assumption (offset of zero), reproduced the complete-case estimate closely (*B* = 0.0028, *SE* = 0.0011, *p* = 0.007, fraction of missing information 0.05). We then added a fixed offset to the imputed scores of excluded participants and varied it across the full width of the PHQ-4 scale. The interaction remained statistically significant at every offset from −12 to +12 points, so no tipping point exists within the range of possible scores, but its statistical support declines steadily as the offset grows: *p* = 0.020 at an offset of +4 points, *p* = 0.040 at +8 points, and *p* = 0.049 at the maximum offset of +12 points. Negative offsets strengthened the estimate (*p* = 0.004 at −4 points). Inverse probability weighting, using a logistic model of the probability of complete data, produced an attenuated estimate that fell marginally short of conventional significance (*B* = 0.0026, robust *SE* = 0.0014, *p* = 0.050, 95% CI −0.0000 to 0.0053). Taken together, the interaction is not an artifact of the excluded cases, but the inverse-probability-weighted result and the behavior of the delta series both remind us that its statistical support is not large.

Finally, an exploratory test of whether the moderation differed by sex was significant (sex × support × self-stigma *B* = −0.009, *p* = 0.003; block of three sex interactions *F*(3, 447) = 3.75, *p* = 0.011). In sex-stratified models, the interaction was present among female students (*n* = 369; *B* = 0.0046, *p* < 0.001) and not detected among male students (*n* = 89; *B* = −0.0041, *p* = 0.101). Because only 89 male students had complete data, this stratified comparison is underpowered, and no test of measurement invariance by sex was possible; the difference is reported for completeness and should be treated as hypothesis-generating.

## 4. Discussion

### 4.1. Principal Findings

In this cross-sectional study of Kuwait University students, the inverse association between perceived social support and psychological distress varied according to levels of help-seeking self-stigma. Higher perceived social support was associated with lower psychological distress among students reporting low self-stigma, whereas this association became progressively weaker as self-stigma increased and was not statistically distinguishable from zero at high levels of self-stigma. H1 was supported: perceived social support was inversely associated with distress. H2 was not supported: help-seeking self-stigma showed no independent association with distress, either bivariately or in the adjusted model. The exploratory moderation was detected and survived an extensive set of sensitivity analyses, but four features of the result constrain how it should be read. The effect is small (Δ*R*^2^ = 0.015; *f*^2^ = 0.016). Its inference weakens appreciably under heteroscedasticity-consistent estimation (*p* = 0.040) and under inverse probability weighting (*p* = 0.050). The interaction is crossover rather than a uniform attenuation of a protective association and cannot be summarized as higher stigma weakening the benefit of support: expressed in PHQ-4 points (Table 7 and Section 3.9), self-stigma level is associated with a statistically detectable difference in distress only at high perceived support, and the point estimate reverses sign at low support. And, most importantly, it is carried entirely by half of a scale that does not behave unidimensionally in this population.

### 4.2. Measurement of Help-Seeking Self-Stigma in This Sample

The confirmatory factor analysis produced the most consequential finding of this study. The ten scored SSOSH items did not form a single dimension; instead, they separated cleanly by item wording, and the two resulting composites were statistically unrelated (*r* = −0.01). This pattern is well documented for mixed-worded instruments. Negatively or reverse-worded items routinely generate a method factor distinct from the substantive trait (DiStefano & Motl, 2006), a pattern first shown clearly for self-esteem measures to be an artifact of wording rather than a substantive second dimension (Marsh, 1996). The mechanism is generally attributed to acquiescent responding combined with the greater cognitive difficulty of verifying a negated statement (Weijters & Baumgartner, 2012; Weijters et al., 2013), and reverse wording appears to create more measurement problems than it solves (van Sonderen et al., 2013). Critically, the problem is not culturally neutral: reverse-worded items degrade measurement more severely outside Western samples (Wong et al., 2003), and in a large sample of United Arab Emirates university students, the factor structure of an Arabic-administered mixed-worded scale was best represented as unidimensional with wording-specific method factors rather than as two substantive dimensions (Dodeen, 2015). Our finding is therefore consistent with an established measurement phenomenon in Arabic-speaking student populations rather than an isolated anomaly, and it also explains the previously unexplained behavior of item 10 and the failure of the congeneric model to yield an interpretable ω.

What makes this more than a technical footnote is that the two composites relate to the other study variables in opposite directions, and that the moderation is carried entirely by the reverse-worded set. The positively worded items behave as a measure of self-stigma should: they are associated with greater psychological distress and, strongly, with greater perceived barriers to care (*r* = 0.40). Yet they show no interaction with perceived social support. The reverse-worded items, which drive the interaction, are associated with lower perceived social support but are unrelated to barriers to care. At least two readings are possible. The reverse-worded composite may capture something closer to a general openness to being helped, or a generalized positive appraisal of oneself and one’s relationships, which would plausibly interact with perceived support. Alternatively, and less interestingly, both the reverse-worded composite and the MSPSS are positively worded agreement-format scales, so their shared association may partly reflect a common response style, and the interaction may be a product of that shared style rather than of self-stigma. The present data cannot distinguish these accounts. What can be said is that the moderation reported here should not be described as a moderation by help-seeking self-stigma as that construct is conventionally understood, and that the finding requires replication with a properly validated Arabic instrument before it is interpreted substantively.

This has a direct implication for future work in the region. No peer-reviewed psychometric validation of an Arabic SSOSH currently exists, and our results indicate that one is needed before the scale is used as a total score in Arabic-speaking samples. A validation study should explicitly test unidimensional, wording-method-factor, and two-factor models, and should examine measurement invariance across sex.

One further caution about scoring follows. The interaction coefficient is 0.0029 with the ten-item score and 0.0037 with the nine-item score, and both are statistically significant. Concordant significance should not be read as evidence that the two operationalizations are equivalent: they differ in scale range and unit, the nine-item score removes an item that our analysis places with the positively worded block, and the difference in magnitude is itself a consequence of the structure documented above. Their agreement indicates that the finding is not an artifact of item 10 alone; it does not indicate that the two scores measure the same thing.

### 4.3. Interpretation of the Moderation

With that qualification in place, the interaction pattern is interpretable. Perceived social support reflects an individual’s belief that supportive relationships are available rather than the amount of support that is actually received or used, and the two are only modestly correlated (Haber et al., 2007; Uchino, 2009). Contemporary accounts hold that perceived support reduces distress through ordinary relational affect regulation (Lakey & Orehek, 2011) and through interpersonal emotion regulation that requires disclosure (Marroquín, 2011), and that received support translates into benefit mainly when it is actually mobilized in the presence of need (Melrose et al., 2015). Each of these mechanisms can be interrupted by a reluctance to acknowledge or disclose difficulty. There is direct evidence that stigma surrounding emotional distress inhibits support-seeking within close relationships specifically, so that available support goes unused (Bishop & High, 2025). A student who believes support is available, but who cannot bring themselves to draw on it would show precisely the pattern observed here: an intact perception of support, and no association between that perception and distress.

We want to be exact about the status of that account. What the data show is heterogeneity: the association between perceived support and distress differs across levels of a self-report measure of help-seeking self-stigma. The data do not show that self-stigma diminishes the psychological benefit of support, because neither disclosure nor use of support was measured, the design is cross-sectional, and the interaction is crossover in form. The defensible statement, and the one we use throughout, is that help-seeking self-stigma functions here as a contextual marker associated with variation in the support–distress relationship rather than as an agent acting upon it. The mechanistic account above is offered as a hypothesis for future testing, not as an interpretation of the present result.

Whether professional help-seeking self-stigma indexes such a general reluctance remains genuinely unsettled, and the evidence is conflicting. Pattyn et al. (2014) found that self-stigma was associated with devaluing formal care but not with attitudes toward informal help-seeking, which would argue against generalization; Kelly and Tonge (2025) found that internalized stigma predicted lower willingness to seek help from friends and family for personal and emotional problems, which would argue for it. Related constructs make generalization plausible: self-concealment is associated with both greater distress and avoidance of help (Cepeda-Benito & Short, 1998; Larson et al., 2015), and anticipated risk of disclosure predicts help-seeking intentions (Vogel & Wester, 2003). In Gulf students specifically, self-stigma, loss of face, and self-disclosure jointly shape help-seeking attitudes (Heath et al., 2016). We did not measure disclosure, received support, or informal help-seeking, so we cannot adjudicate between these positions. We therefore treat this as an open empirical question with evidence on both sides, rather than as an explanation of our finding.

An important feature of the findings is that help-seeking self-stigma was not independently associated with psychological distress at the bivariate level or in the adjusted regression model. This pattern is not contradictory: a variable may modify the association between an exposure and an outcome without demonstrating a main effect. It is, however, notable that the null total-score association is itself a consequence of the two-factor structure, since the positively worded and reverse-worded components correlate with distress at 0.25 and −0.16, respectively, and therefore cancel. This is a further reason to treat the total score with caution.

Several alternative explanations should be considered. The interaction may partly reflect sampling variability, the exploratory nature of the analysis, differential missing data, response-style variance shared between the reverse-worded SSOSH items and the MSPSS, or sparse observations at some combinations of perceived social support and self-stigma. Because the study was cross-sectional, the temporal ordering of these constructs cannot be established, and the findings should not be interpreted as evidence that self-stigma reduces the effectiveness of social support.

### 4.4. Relation to Previous Literature

The inverse association between perceived social support and psychological distress observed in this study is consistent with a substantial body of the literature among university students (Ruihua et al., 2025; Vicary et al., 2025) and with the stress-buffering framework (S. Cohen & Wills, 1985). However, the present study should be viewed as broadly consistent with that framework rather than a direct test, because it did not examine a stressor-by-support interaction and its cross-sectional design precludes causal inference.

The observation that family support showed the strongest association with distress is consistent with evidence from Arab adolescent and student samples, where family is typically the dominant source of support and the strongest correlate of mental health (Alshammari et al., 2021; El-Haj-Mohamad et al., 2025). It is also consistent with the three-factor structure of the Arabic MSPSS reported by Fekih-Romdhane et al. (2023a), which our confirmatory analysis reproduced. The high intercorrelation of the three subscales in our data (0.64 to 0.72) nonetheless limits how much weight can be placed on differences between them.

Previous research has consistently identified help-seeking self-stigma as an important barrier to professional mental health service utilization, with meta-analytic evidence indicating that personal or internalized stigma, rather than perceived public stigma, is the component associated with reduced active help-seeking (Schnyder et al., 2017; Yu et al., 2023). Studies conducted in Arab and Gulf countries similarly indicate that concerns related to family reputation, anticipated social judgment, and perceived shame contribute to help-seeking stigma (Al-Krenawi et al., 2009; El-Shamy et al., 2023), and recent regional work documents continuing high levels of public stigma (Alhamad et al., 2026; Alshawwa & Caldwell-Harris, 2026) and a preference among Gulf students for informal over professional sources of help (Aldhamri et al., 2026).

Work closest to the present finding concerns moderation of the support–distress association by person-level characteristics. Meta-analytic evidence establishes that this association is systematically conditional (Rueger et al., 2016), and specific moderators have been identified, including attachment style (Costa-Cordella et al., 2022; Dark-Freudeman et al., 2020) and the capacity to receive compassion from others, which buffers the depressogenic effect of self-criticism (Hermanto et al., 2016). The closest direct precedent is Sabado-Liwag et al. (2025), who reported that mental-health self-stigma moderated the association between social support and depression, anxiety, and stress among Pacific Islander young adults, with family support again prominent. Our results are broadly consistent with the literature in showing that an internal, self-evaluative characteristic conditions whether perceived support is associated with lower distress, while adding the cautionary observation that measuring that characteristic is itself fragile.

### 4.5. Sex Differences

Male students had lower adjusted PHQ-4 scores than female students, consistent with the well-established female preponderance in depressive and anxiety symptoms in student and general populations (Auerbach et al., 2018; Salk et al., 2017). Whether such differences reflect true differences in symptom experience or differences in willingness to report them remains contested (Sigmon et al., 2005), and men’s lower endorsement of distress coexists with less favorable help-seeking attitudes and greater reluctance to disclose (Nam et al., 2010; Sagar-Ouriaghli et al., 2019). Our exploratory analysis further suggested that the moderation itself was present among female students and not detected among male students. With only 89 male students in the complete-case sample, this comparison is underpowered, and no test of measurement invariance across sex was possible; given the wording-related measurement problems documented in Section 3.2, apparent sex differences could reflect differential item functioning as easily as substantive differences. The finding is reported to avoid selective reporting and should not be interpreted as evidence that male students experience better mental health.

### 4.6. Practical Implications

Before any implication is drawn, one distinction should be stated plainly, because the volume of sensitivity analysis reported here could otherwise be mistaken for confirmation. Showing that the same interaction persists across alternative analyses of the same dataset establishes that it is not an artifact of one modeling decision, one estimator, or a few influential cases. It does not convert a data-identified exploratory interaction into a confirmatory finding, because each of those analyses reuses the data in which the interaction was found. Robustness and confirmation are different properties, and only the first is demonstrated here.

The present findings do not establish that reducing help-seeking self-stigma would strengthen the association between perceived social support and psychological well-being, and this cross-sectional study did not evaluate any intervention. Any implication is therefore preliminary and conditional on replication. That said, the pattern is consistent with a broader observation in the intervention literature: interventions that address maladaptive social cognition outperform those that simply increase social opportunity (Masi et al., 2011). If replicated with a validated instrument and in a longitudinal design, our findings would justify testing whether initiatives that build supportive campus environments (Ellard et al., 2023; Pointon-Haas et al., 2024) are more effective when combined with components that address negative self-evaluations about needing help. Both halves of that combination have some evidence behind them: stigma-reduction interventions in educational settings can improve stigma-related outcomes (Ma et al., 2023; Waqas et al., 2020), contact-based approaches have the strongest support in the general literature though with limited evidence of long-term effect (Thornicroft et al., 2016), universal campus prevention programs produce small but real benefits (Conley et al., 2015), and interventions promoting help-seeking show modest effects (Xu et al., 2018). Whether combining them yields more than either alone remains untested.

A more immediate implication concerns measurement. Services and researchers in Kuwait and the wider Gulf who wish to measure help-seeking self-stigma should not assume that a translated mixed-worded scale performs as it does in the source population. On the evidence presented here, a total SSOSH score would misrepresent the construct in this population.

### 4.7. Strengths and Limitations

This study has several strengths. It used established measures, reported sample-specific reliability and item performance, and evaluated the exploratory interaction through a wide range of complementary sensitivity analyses, including robust standard errors, multiple imputation with a delta-adjusted sensitivity analysis, inverse probability weighting, bootstrap estimation, leave-one-out analysis, alternative scale scorings, a generalized linear model appropriate to the outcome distribution, and adjustment for perceived barriers to care. It reports the exploratory status of the moderation transparently and reports a measurement problem that weakens its headline claim rather than omitting it.

Several limitations must be acknowledged. First, and most importantly, the moderator’s measurement is compromised. The Arabic SSOSH was not unidimensional in this sample; the moderation is carried by the reverse-worded items alone, and the construct those items measure in this population is uncertain. Accordingly, the central finding should be treated as provisional.

Second, the cross-sectional design precludes causal inference, and the observed interaction reflects differences in associations rather than evidence of an underlying mechanism. The convenience sample was drawn from a single university, was predominantly female, and had an unknown response rate. Because recruitment used open channels, the number of students who saw the invitation was not recorded, so no response rate can be computed. Regarding the sex composition, Kuwait University does not publish a routine sex-disaggregated enrolment table; the most recent institution-level figures indicate a student body of approximately 29,943 with a female-to-male ratio of about 73:27 (Times Higher Education, 2026), and the University’s own graduate statistics for 2022/2023 record 4784 female (77.3%) and 1407 male (22.7%) graduates (Kuwait University, 2024). Our sample, at 80.9% female, is therefore somewhat more female-skewed than the university population, but the university itself is strongly female-majority, which bounds the magnitude of this particular selection bias. It does not bound others: students willing to complete a mental-health survey may differ systematically from those who are not, in ways we cannot assess here.

Third, all measures were self-reported and administered at a single time point. Statistical diagnostics did not indicate a dominant method factor, and common method variance cannot produce a spurious interaction (Siemsen et al., 2010), but shared response style between the reverse-worded SSOSH items and the MSPSS remains a live alternative explanation for the specific interaction observed. The study did not assess emotional disclosure, received informal support, or whether perceived support was actually used, so the mechanisms underlying the interaction could not be evaluated directly.

Fourth, the moderation analysis was exploratory, not preregistered, conducted after inspection of the data, and not adjusted for multiple testing; the sensitivity analyses were developed after the interaction was identified. Findings should be regarded as hypothesis-generating and require confirmation in independent preregistered studies.

Fifth, the interaction explained a small proportion of variance. This is not unusual: a 30-year review of moderator tests found that observed interaction effects in field research are typically very small and that such tests are chronically underpowered (Aguinis et al., 2005; see also Chaplin, 1991), so an effect of this size is neither anomalous nor self-evidently trivial. It does, however, mean that the practical reach of the finding is limited, a point reinforced by the Johnson–Neyman analysis: the region in which the conditional association is statistically detectable covers only about 58 to 64% of the sample, so for a substantial minority of students no conclusion about the support–distress association can be drawn from this model. No a priori power analysis was conducted for the interaction; with *n* = 458 and seven predictors, the study had approximately 80% power to detect an interaction of *f*^2^ = 0.017, which is close to the effect actually observed, so the analysis was adequately, though not generously, powered.

Finally, the online questionnaire inadvertently allowed ambiguous multiple responses to PHQ-4 items, resulting in the exclusion of 49 participants from the complete-case analysis; these participants differed on the moderator. Multiple imputation, delta-adjusted sensitivity analysis, and inverse probability weighting all produced estimates close to the complete-case estimate, but each depends on assumptions, and none can fully overcome the original questionnaire design limitation. Future administrations should use a forced-choice response format with validation.

## 5. Conclusions

In this cross-sectional study of Kuwait University students, the inverse association between perceived social support and psychological distress differed according to levels of help-seeking self-stigma, and the interaction was consistent across an extensive set of sensitivity analyses. Two qualifications are essential. The effect was small, and its statistical support weakened under heteroscedasticity-consistent and inverse-probability-weighted estimation. More fundamentally, the Arabic Self-Stigma of Seeking Help Scale was not unidimensional in this sample, and the interaction was carried entirely by its reverse-worded items, whose construct meaning in this population is uncertain. The finding is therefore best read as an indication that some self-evaluative or response-related characteristic conditions the association between perceived support and distress, and as a demonstration that measuring help-seeking self-stigma in Arabic-speaking student populations requires attention before substantive conclusions are drawn. Psychometric validation of an Arabic SSOSH, followed by preregistered longitudinal and intervention studies, is needed to determine whether help-seeking self-stigma meaningfully influences the relationship between perceived social support and psychological distress over time.

## Figures and Tables

**Table 1:** Participant characteristics (*N* = 507).

Characteristic	*n* (%) or *M* (*SD*)
***Sex***	
Female	410 (80.9)
Male	97 (19.1)
Age, years	21.44 (4.38); range 18–45
***Nationality***	
Kuwaiti	435 (85.8)
Non-Kuwaiti	55 (10.8)
Treated-as-Kuwaiti	17 (3.4)
***Year of study***	
First year	106 (20.9)
Second year	137 (27.0)
Third year	124 (24.5)
Fourth year or higher	131 (25.8)
Graduate studies	9 (1.8)
***College type***	
Health sciences	174 (34.3)
Non-health sciences	329 (64.9)
Not reported	4 (0.8)
Grade point average (of 4.00)	3.13 (0.58); *n* = 494

Percentages may not total 100 because of rounding. “Treated-as-Kuwaiti” is an administrative category used by Kuwait University for students who are not Kuwaiti nationals but are admitted and charged fees on the same basis as Kuwaiti nationals; it principally comprises children of Kuwaiti mothers and non-Kuwaiti fathers, children of GCC nationals, and certain long-term residents. Because this group is small and administratively similar to other non-Kuwaiti students, it was combined with non-Kuwaiti students for analysis.

**Table 2:** Descriptive statistics and internal consistency of the study measures.

Measure (Possible Range)	*n*	*M* (*SD*)	Observed Range	α	ω
PHQ-4 total (0–12)	458	5.13 (3.24)	0–12	0.83	0.83
GAD-2 anxiety (0–6)	480	2.73 (1.83)	0–6	—	—
PHQ-2 depression (0–6)	478	2.38 (1.77)	0–6	—	—
MSPSS total (12–84)	507	54.19 (20.28)	12–84	0.95	0.95
Significant other (1–7)	507	4.78 (1.93)	1–7	0.94	0.94
Family (1–7)	507	4.43 (1.87)	1–7	0.92	0.92
Friends (1–7)	507	4.34 (1.91)	1–7	0.94	0.94
SSOSH total (10–50)	507	25.07 (6.58)	10–49	0.72	0.61 a
Positively worded, 5 items (5–25)	507	10.59 (4.27)	5–25	0.82	0.82
Reverse worded, 5 items (5–25)	507	14.48 (5.05)	5–25	0.82	0.82
BACE-30 total (0–90)	507	30.83 (18.33)	0–90	0.94	0.94

a ω for the SSOSH total is reported for completeness but should not be interpreted, because the single-factor model on which it depends did not fit the data (Section 3.2). MSPSS subscale scores are item means, so their possible range is 1–7. Reliability coefficients for the two-item PHQ-4 subscales are not reported because α is not informative for two-item scales.

**Table 3:** Zero-order correlations among the study variables.

	(1)	(2)	(3)	(4)	(5)	(6)	(7)	(8)	(9)	(10)
(1) PHQ-4 total	—									
(2) GAD-2 anxiety	0.91 ***	—								
(3) PHQ-2 depression	0.90 ***	0.63 ***	—							
(4) MSPSS total	−0.16 ***	−0.06	−0.19 ***	—						
(5) MSPSS significant other	−0.13 **	−0.04	−0.15 **	0.91 ***	—					
(6) MSPSS family	−0.19 ***	−0.09 *	−0.22 ***	0.87 ***	0.70 ***	—				
(7) MSPSS friends	−0.10 *	−0.03	−0.14 **	0.88 ***	0.72 ***	0.64 ***	—			
(8) SSOSH total	0.05	0.01	0.02	−0.28 ***	−0.28 ***	−0.22 ***	−0.24 ***	—		
(9) SSOSH positively worded	0.25 ***	0.22 ***	0.22 ***	−0.06	−0.06	−0.05	−0.04	0.64 ***	—	
(10) SSOSH reverse worded	−0.16 ***	−0.16 ***	−0.16 ***	−0.31 ***	−0.32 ***	−0.25 ***	−0.27 ***	0.76 ***	−0.01	—
(11) BACE-30 total	0.27 ***	0.23 ***	0.24 ***	−0.05	−0.00	−0.09 *	−0.04	0.22 ***	0.40 ***	−0.05

*n* = 458 for PHQ-4 total, 480 for GAD-2, 478 for PHQ-2, and 507 for all other variables. * *p* < 0.05, ** *p* < 0.01, *** *p* < 0.001.

**Table 4:** Hierarchical linear regression predicting psychological distress (PHQ-4 total score), *n* = 458.

Predictor	*B*	*SE*	95% CI	β	*t*	*p*
***Block 1 (R*^2^* = 0.026)***						
Age (years)	0.009	0.039	−0.067 to 0.084	0.01	0.23	0.821
Sex (male vs. female)	−1.155	0.385	−1.911 to −0.399	−0.14	−3.00	0.003
Nationality (non-Kuwaiti vs. Kuwaiti)	0.677	0.422	−0.153 to 1.507	0.07	1.60	0.110
Year of study (3rd+ vs. 1st–2nd)	−0.088	0.332	−0.741 to 0.564	−0.01	−0.27	0.790
***Block 2 (R*^2^* = 0.058, *Δ*R*^2^* = 0.032)***						
Perceived social support (centered)	−0.025	0.008	−0.041 to −0.010	−0.16	−3.28	0.001
Help-seeking self-stigma (centered)	0.028	0.024	−0.020 to 0.076	0.06	1.15	0.250
***Block 3—final model (R*^2^* = 0.073, *Δ*R*^2^* = 0.015)***						
Age (years)	−0.003	0.038	−0.078 to 0.071	−0.00	−0.09	0.931
Sex (male vs. female)	−1.312	0.391	−2.081 to −0.543	−0.16	−3.35	0.001
Nationality (non-Kuwaiti vs. Kuwaiti)	0.647	0.416	−0.171 to 1.464	0.07	1.56	0.121
Year of study (3rd+ vs. 1st–2nd)	0.053	0.327	−0.589 to 0.695	0.01	0.16	0.871
Perceived social support (centered)	−0.026	0.008	−0.041 to −0.011	−0.16	−3.41	0.001
Help-seeking self-stigma (centered)	0.025	0.024	−0.023 to 0.073	0.05	1.03	0.305
Support × self-stigma	0.003	0.001	0.001 to 0.005	0.12	2.70	0.007

Constant = 5.459 (95% CI 3.914 to 7.005). Final model: *F*(7, 450) = 5.04, *p* < 0.001; *R*^2^ = 0.073, adjusted *R*^2^ = 0.058. Interaction block: Δ*R*^2^ = 0.015, *F*(1, 450) = 7.27, *p* = 0.007, *f*^2^ = 0.016. All variance inflation factors ≤ 1.24. Sex was coded 0 = female, 1 = male; nationality 0 = Kuwaiti, 1 = non-Kuwaiti or treated-as-Kuwaiti; year of study 0 = first or second year, 1 = third year or higher. Because the predictors are mean-centered, the coefficient for perceived social support is its conditional slope at the centering value of self-stigma, that is, at the full-sample mean of 25.07; Section 3.9 probes the same slope at the complete-case mean of 24.87, which is why the two values differ slightly (−0.026 versus −0.027). Centering affects only the lower-order coefficients and the constant; the interaction estimate, its standard error and its *p*-value are identical under any centering. This table reports the primary model only; models additionally adjusted for barriers to care are reported in Section 3.10.

**Table 5:** The perceived support × self-stigma interaction estimated with each operationalization of help-seeking self-stigma (*n* = 458).

Moderator	*B*	*SE*	*t*	*p*	HC3 *p*	Δ*R*^2^
SSOSH total, 10 items (primary)	0.0029	0.0011	2.70	0.007	0.040	0.015
SSOSH 9 items (item 10 excluded)	0.0037	0.0011	3.42	0.001	0.008	0.024
Reverse-worded composite, 5 items	0.0049	0.0012	4.00	<0.001	<0.001	0.031
Positively worded composite, 5 items	−0.0022	0.0016	−1.35	0.177	0.203	0.004
Positively worded composite, 4 items	−0.0012	0.0020	−0.60	0.550	0.573	0.001

Each row is a separate model in which the stated composite replaces the SSOSH total score as both the main effect and the constituent of the interaction term. All models adjust for age, sex, nationality, year of study, and perceived social support. HC3 *p*-values are from the same models estimated with heteroscedasticity-consistent standard errors; the ordering of the five operationalizations is unchanged under robust estimation.

**Table 6:** Association of perceived social support with psychological distress at three levels of help-seeking self-stigma.

Level of Help-Seeking Self-Stigma	SSOSH Score	*B*	95% CI	*p*	HC3 *p*
Low (−1 *SD*)	18.26	−0.046	−0.067 to −0.025	<0.001	<0.001
Mean	24.87	−0.027	−0.042 to −0.012	<0.001	0.002
High (+1 *SD*)	31.48	−0.008	−0.027 to 0.012	0.454	0.565

Estimates are adjusted for age, sex, nationality, and year of study. HC3 *p*-values are from the same model estimated with heteroscedasticity-consistent standard errors.

**Table 7:** Predicted difference in PHQ-4 score associated with higher perceived social support, by level of help-seeking self-stigma.

Level of Help-Seeking Self-Stigma	10th to 90th Percentile of Support (24 to 80)	Interquartile Range of Support (42 to 72)
Low (−1 *SD*, SSOSH 18.26)	−2.58 [−3.78, −1.38]	−1.38 [−2.02, −0.74]
Mean (SSOSH 24.87)	−1.50 [−2.35, −0.65]	−0.80 [−1.26, −0.35]
High (+1 *SD*, SSOSH 31.48)	−0.42 [−1.54, 0.69]	−0.23 [−0.82, 0.37]

Values are predicted differences in PHQ-4 total score with 95% confidence intervals, adjusted for age, sex, nationality, and year of study. Negative values indicate lower predicted distress at higher perceived social support. For reference, the observed PHQ-4 mean was 5.13, and the standard deviation was 3.24.

**Table 8:** Subscale analyses: interaction of perceived social support with help-seeking self-stigma across PHQ-4 components and MSPSS components.

Model	*n*	*B*	*SE*	*t*	*p*	HC3 *p*	Δ*R*^2^
***Outcome (predictor = MSPSS total)***							
PHQ-4 total	458	0.0029	0.0011	2.70	0.007	0.040	0.015
GAD-2 anxiety	480	0.0019	0.0006	3.15	0.002	0.010	0.020
PHQ-2 depression	478	0.0012	0.0006	2.04	0.042	0.127	0.008
GAD-2 anxiety, restricted to *n* = 458	458	0.0018	0.0006	3.01	0.003	0.012	0.019
PHQ-2 depression, restricted to *n* = 458	458	0.0011	0.0006	1.83	0.068	0.169	0.007
***Predictor (outcome = PHQ-4 total)***							
MSPSS significant other	458	0.0210	0.0112	1.87	0.062	0.135	0.007
MSPSS family	458	0.0328	0.0120	2.73	0.007	0.021	0.015
MSPSS friends	458	0.0223	0.0110	2.04	0.043	0.093	0.009

All models adjust for age, sex, nationality, and year of study, and include the main effects of the relevant support measure and of help-seeking self-stigma. MSPSS subscale scores are item means (range 1–7), so their coefficients are not directly comparable in magnitude with those for the MSPSS total score (range 12–84). The two restricted rows re-estimate the subscale outcomes on the 458 participants with complete data on all four PHQ-4 items, to show the sensitivity of each result to the scoring rule described in Section 2.3. The three MSPSS subscale models are estimated separately, one subscale at a time; entered simultaneously, none of the three interactions is individually significant, and the block as a whole is marginal (Δ*R*^2^ = 0.017, *F*(3, 446) = 2.72, *p* = 0.044).

**Table 9:** Sensitivity and robustness analyses for the perceived support × self-stigma interaction.

Analysis	*B*	*SE* or 95% CI	*p*	HC3 *p*
Primary model (OLS)	0.0029	0.0011	0.007	0.040
BCa bootstrap, 5000 resamples	0.0029	0.0002 to 0.0056	—	—
Leave-one-out (458 models)	0.0027 to 0.0036	—	always positive	—
Excluding cases flagged by Cook’s D > 4/*n* (*n* = 434)	0.0051	0.0011	<0.001	—
Nine-item SSOSH (item 10 excluded)	0.0037	0.0011	0.001	0.008
Reverse-worded composite as moderator	0.0049	0.0012	<0.001	<0.001
Positively worded composite as moderator	−0.0022	0.0016	0.177	0.203
Additionally adjusted for BACE-30	0.0027	0.0010	0.010	0.036
Poisson GLM, robust standard errors	0.0006	0.0003	0.022	—
Multiple imputation, item level, 50 datasets	0.0032	0.0011	0.003	—
Re-specified imputation, offset 0 (MAR)	0.0028	0.0011	0.007	—
Delta-adjusted imputation, offset +4 points	0.0026	0.0011	0.020	—
Delta-adjusted imputation, offset +12 points	0.0023	0.0012	0.049	—
Inverse probability weighting	0.0026	0.0014	0.050	—

The HC3 column reports the same model re-estimated with heteroscedasticity-consistent standard errors; a dash indicates that the analysis already uses a robust, resampled, or pooled variance estimator, so a separate HC3 estimate is not applicable. No *p*-value is calculated for the bootstrap; inference is based on the confidence interval. The delta-adjusted rows show representative offsets; the interaction remained statistically significant at every offset between −12 and +12 PHQ-4 points, with *p* rising monotonically from 0.007 at an offset of zero to 0.049 at +12 points.

## Data Availability

The de-identified data supporting the findings are available from the corresponding author upon reasonable request and subject to approval by Kuwait University and any applicable ethical or institutional data-sharing requirements. The data are not publicly deposited because the participant consent process did not include unrestricted public data sharing.

## Supplementary Materials

The following supporting information can be downloaded at: https://www.mdpi.com/article/10.3390/ejihpe16090133/s1, Table S1: Completed reporting checklist based on Turk et al. (2018). Table S2: SSOSH item-level statistics. Table S3: Confirmatory factor analysis fit indices for the SSOSH and the MSPSS. Table S4: Common method variance diagnostics. Table S5: Regression assumption and influence diagnostics. Table S6: Comparison of included and excluded participants. Figure S1: SSOSH item correlation matrix after reverse scoring. Figure S2: Adjusted PHQ-4 predictions by social support and self-stigma, expanded vertical axis.

## Abbreviations

BACE-30: Barriers to Access to Care Evaluation (30-item); BCa: Bias-corrected and accelerated; CFA: Confirmatory factor analysis; CFI: Comparative fit index; CI: Confidence interval; GAD-2: Generalized Anxiety Disorder-2; HC3: Heteroscedasticity-consistent standard error estimator (type 3); IPW: Inverse probability weighting; MSPSS: Multidimensional Scale of Perceived Social Support; OLS: Ordinary least squares; PHQ-2: Patient Health Questionnaire-2; PHQ-4: Patient Health Questionnaire-4; RMSEA: Root mean square error of approximation; SRMR: Standardized root mean square residual; SSOSH: Self-Stigma of Seeking Help Scale; TLI: Tucker–Lewis index; VIF: Variance inflation factor.
