# Supplementary material for: Help-Seeking Self-Stigma and the Association Between Perceived Social Support and Psychological Distress Among Kuwait University Students: An Exploratory Cross-Sectional Moderation Analysis
Source: Eur J Investig Health Psychol Educ. 2026 Sep 2;16(9):133. doi: 10.3390/ejihpe16090133 (PMC13605599; doi:10.3390/ejihpe16090133)
Supplement: Supplementary file 1 [file ejihpe-16-00133-s001.zip › ejihpe-4500765-supplementary.pdf]

Supplementary Material

Help-Seeking Self-Stigma and the Association Between Perceived Social Support and Psychological Distress Among Kuwait University Students

Fatima Al-Ghadban and Ahmad Salman

Table S1. Reporting checklist for web-based survey studies, based on Turk et al. (2018).

The checklist below follows the items applied by Turk et al. (2018), which combine the SUrvey Reporting GuidelinE (SURGE) for survey studies generally with the Checklist for Reporting Results of Internet E-Surveys (CHERRIES) for web-based surveys. Section numbers refer to the revised manuscript.

Item	Reported	Where / detail
Design and reporting		
Study design stated in title and abstract	Yes	Title and Abstract
Background provided	Yes	Section 1
Purpose or aim explicitly stated	Yes	Section 1, final three paragraphs
Hypotheses stated	Yes	Section 1 (H1, H2, and the exploratory question)
Target population, sample frame and convenience sampling described	Yes	Sections 2.1 and 2.2
Research tool		
Description of the questionnaire	Yes	Section 2.3; example items for every subscale
Existing tools: references to original work	Yes	Section 2.3
Existing tools: psychometric properties presented	Yes	Sections 2.3 and 3.1–3.2; Tables 2, S2, S3
Scoring procedures described	Yes	Section 2.3
Development and pre-testing of the instrument	Partly	Section 2.1: no separate pilot or cognitive interviewing was conducted; stated as a limitation in Section 4.7
Sample selection		
Description of representativeness of the sample	Yes	Section 4.7, including comparison with university sex distribution
Sample size calculation or rationale	Partly	Section 2.2: no a priori calculation; post hoc detectable effect size in Section 4.7
Survey administration		
Mode of administration	Yes	Section 2.1: self-administered online questionnaire (Google Forms)
Open versus closed survey	Yes	Section 2.1: open survey; no password or registration
Contact mode	Yes	Section 2.1: university email announcements, student communication platforms, college networks
Advertising the survey	Yes	Section 2.1
Wording of the advertisement	No	Not recorded
Context of the platform on which the survey was posted	Partly	Section 2.1
Mandatory or voluntary	Yes	Section 2.1: voluntary
Incentives	Yes	Section 2.1: none offered
Time frame of data collection	Yes	Section 2.1: February to April 2026
Randomisation of items	Yes	Section 2.1: not randomised
Adaptive questioning	Yes	Section 2.1: used only for routing
Number of items per page and number of screens	Yes	Section 2.1: 11 screens
Completeness check before submission	No	Not implemented; this is the origin of the ambiguous PHQ-4 responses (Sections 2.3 and 4.7)
Non-response option offered	No	Not offered
Review step allowing respondents to change answers	Yes	Section 2.1

Item	Reported	Where / detail
Response rates		
Unique site visitors	No	Not captured (open recruitment)
View rate	Not calculable	Denominator unknown; stated in Sections 2.1 and 4.7
Participation rate	Not calculable	Denominator unknown
Completion rate	Yes	543 questionnaires submitted; 507 (93.4%) eligible; 458 (84.3% of submitted, 90.3% of eligible) had complete PHQ-4 data
Preventing multiple entries		
Cookies, IP check, or log-file analysis used	No	Section 2.1; acknowledged as a limitation
Analysis		
Method of data analysis described	Yes	Section 2.5
Handling of incomplete questionnaires	Yes	Sections 2.3, 2.5.6, 3.4 and 3.10
Method for handling item-missing data	Yes	Section 2.5.6: complete-case, multiple imputation, delta-adjusted sensitivity analysis, inverse probability weighting
Analysis of non-response error	Yes	Section 3.4; Table S6
Statistical correction for a non-representative sample	Yes	Inverse probability weighting, Section 3.10
Atypical timestamps	No	Not examined
Results and discussion		
All respondents accounted for	Yes	Section 2.2 and Section 3.1
Results clearly presented and addressing objectives	Yes	Section 3
Strengths stated	Yes	Section 4.7
Limitations stated	Yes	Section 4.7
Generalisability discussed	Yes	Section 4.7
Ethics		
Ethics committee review reported	Yes	Section 2.6 and Declarations; approval date given
Informed consent process reported	Yes	Section 2.2 and 2.6
Data protection	Yes	Section 2.1: fully anonymous; no personal identifiers collected
Funding reported	Yes	Declarations

Table S2. Item-level statistics for the Self-Stigma of Seeking Help Scale (N = 507).

Item	Wording	M	SD	Skew	Kurtosis	Corrected item–total r	α if deleted
1	Positive	2.07	1.11	0.76	−0.19	0.35	0.706
2	Reverse	2.71	1.38	0.28	−1.13	0.50	0.682
3	Positive	1.93	1.11	1.09	0.45	0.43	0.695
4	Reverse	2.97	1.23	0.13	−0.80	0.40	0.699
5	Reverse	2.95	1.38	0.08	−1.18	0.44	0.692
6	Positive	1.99	1.10	0.83	−0.22	0.45	0.692
7	Reverse	3.07	1.27	0.03	−0.92	0.27	0.720
8	Positive	1.93	1.01	0.89	0.15	0.52	0.685
9	Reverse	2.79	1.38	0.19	−1.15	0.49	0.683
10	Positive	2.67	1.26	0.22	−0.93	0.03	0.756

Item statistics are computed on scored items, that is, after reverse-scoring items 2, 4, 5, 7 and 9. Cronbach's α for the full ten-item scale was 0.72 and for the nine-item scale excluding item 10 was 0.76. Mean inter-item correlation was 0.21 for the ten-item scale and 0.26 for the nine-item scale. Item 10 correlated positively with the other positively worded items (r = 0.29 to 0.41) and negatively with every reverse-worded item (r = −0.14 to −0.29).

Table S3. Confirmatory factor analysis fit indices.

Model	χ^2	df	p	CFI	TLI	RMSEA [90% CI]	SRMR
SSOSH							
One factor, 10 items	931.93	35	<0.001	0.508	0.368	0.225 [0.213, 0.238]	0.215
One factor, 9 items (item 10 removed)	848.78	27	<0.001	0.502	0.337	0.245 [0.231, 0.260]	0.223
Two factors by item wording, 10 items	83.88	26	<0.001	0.968	0.945	0.066 [0.051, 0.082]	0.027
MSPSS							
One factor, 12 items	1790.24	54	<0.001	0.722	0.660	0.252 [0.242, 0.262]	0.091
Three factors, 12 items	257.63	33	<0.001	0.964	0.928	0.116 [0.103, 0.129]	0.020
SSOSH, polychoric correlations							
One factor, 10 items	1291.10	35	<0.001	0.511	0.371	0.266 [0.254, 0.279]	0.245
Two factors by item wording, 10 items	175.00	26	<0.001	0.942	0.900	0.106 [0.092, 0.122]	0.032

Maximum-likelihood estimation on the item correlation matrix, N = 507. Fit was evaluated against the criteria of Hu and Bentler (1999), interpreted as guidelines rather than absolute thresholds (Marsh et al., 2004). Standardised loadings on the single SSOSH factor were 0.46 to 0.83 for the five positively worded items and 0.05 to 0.15 in absolute value for the five reverse-worded items. For the MSPSS, the three-factor solution reproduced the published subscale structure and fitted markedly better than the one-factor solution, although its RMSEA remained above the conventional criterion. The final two rows repeat the SSOSH analysis on the matrix of polychoric correlations, which treats the five-point items as ordinal; the mean polychoric correlation was 0.57 within the positively worded items and 0.54 within the reverse-worded items but 0.01 between them, and the conclusion is unchanged.

Table S4. Common method variance diagnostics.

Diagnostic	Result
Items entered	56 (PHQ-4 4, SSOSH 10, MSPSS 12, BACE-30 30); n = 458
Variance explained by the first unrotated component	22.7%
Components with eigenvalue > 1	11, explaining 67.3% cumulatively
First five eigenvalues	12.71, 8.67, 3.77, 2.80, 1.98
Single common factor model fit	χ^2(1484) = 11,859.2, p < 0.001; CFI = 0.356; TLI = 0.332; RMSEA = 0.124 [0.122, 0.126]; SRMR = 0.164
Variance extracted by the single common factor	21.4%; mean absolute loading 0.39
Focal scales only (26 items: PHQ-4, SSOSH, MSPSS)	First unrotated component explains 32.0%

Harman's single-factor test is an insensitive diagnostic and a null result does not establish the absence of method bias (Fuller et al., 2016; Podsakoff et al., 2003). It is reported alongside the analytical result that common method variance cannot generate spurious interaction effects and instead attenuates true ones (Evans, 1985; Siemsen et al., 2010).

Table S5. Regression assumption and influence diagnostics for the final model (*n* = 458).

Assumption	Test	Statistic	p	Interpretation
Normality of residuals	Shapiro–Wilk	$W = 0.984$	<0.001	Modest departure
	Kolmogorov–Smirnov	D = 0.065	0.041	Modest departure
	Skewness	0.31 ($SE = 0.11$), $z = 2.73$	0.006	Slight positive skew
	Kurtosis	−0.48 ($SE = 0.23$), $z = −2.10$	0.036	Slightly platykurtic
	Jarque–Bera	11.73	0.003	Consistent with the above
Homoscedasticity	Breusch–Pagan (Koenker)	$LM = 24.32$, $df = 7$	0.001	Violated
	White general test	$LM = 78.81$, $df = 31$	<0.001	Violated
	Spearman ρ(|residual|, fitted)	0.135	0.004	Weak but systematic
	Squared std. residual on ZPRED	$F(1, 456) = 2.52$	0.113	Not significant (1 df)
Linearity / functional form	Ramsey RESET (powers 2, 3)	$F(2, 448) = 1.78$	0.170	Adequate
	Quadratic term, social support	$B = −0.00002$, $t = −0.04$	0.965	Not needed
	Quadratic term, self-stigma	$B = 0.008$, $t = 3.16$	0.002	Curvilinear; interaction unaffected
Independence	Durbin–Watson	1.86	—	No concern (cross-sectional data)
Multicollinearity	Variance inflation factors	1.01 to 1.24	—	No concern
	Tolerances	0.81 to 0.99	—	No concern
	Condition number	3.37	—	No concern
Outliers and influence	Standardised residuals	−1.99 to 2.52	—	None exceed |3|
	Studentised residuals	−2.01 to 2.53	—	None exceed |3|
	Cook's distance	max 0.078	—	None exceed 1; 24 exceed $4/n = 0.009$
	Leverage (uncentred hat)	max 0.107	—	33 exceed $2k/n = 0.035$; 15 exceed $3k/n = 0.052$
	Leverage (centred)	max 0.105	—	equals hat − 1/n
	DFBETAS (interaction)	max |0.65|	—	36 exceed 2/√n = 0.094
	Model excluding Cook's D > 4/n	$B = 0.0051$, $SE = 0.0011$, $n = 434$	<0.001	Interaction strengthened

Because the homoscedasticity assumption was not met, HC3 heteroscedasticity-consistent standard errors are reported alongside conventional standard errors throughout the manuscript and should be regarded as the primary inference (Long & Ervin, 2000). Screening thresholds for influence were specified a priori and were used to flag cases for inspection, not for deletion. In the leverage thresholds k denotes the number of estimated parameters including the intercept (k = 8).

Table S6. Comparison of participants included in and excluded from the primary regression analysis.

Variable	Included (n = 458)	Excluded (n = 49)	Test statistic	p	Effect size
Age, years, *M* (SD)	21.35	22.29	$t(55.3) = -1.22$	0.226	$d = -0.21$
MSPSS total, M	54.76	48.84	$t(56.7) = 1.80$	0.078	$d = 0.29$
SSOSH total, M	24.87	26.96	$t(60.8) = -2.27$	0.027	$d = -0.32$
SSOSH 9-item, M	22.19	24.33	$t(61.0) = -2.39$	0.020	$d = -0.33$
SSOSH positively worded, M	10.58	10.71	$t(56.1) = -0.19$	0.848	$d = -0.03$
SSOSH reverse worded, M	14.29	16.24	$t(56.4) = -2.36$	0.022	$d = -0.39$
BACE-30 total, M	30.47	34.20	$t(57.9) = -1.31$	0.195	$d = -0.20$
Grade point average, M	3.13	3.09	$t(55.1) = 0.49$	0.628	$d = 0.08$
Sex	369 F / 89 M	41 F / 8 M	$\chi^2(1) = 0.28$	0.599	V = 0.02
Nationality	390 K / 68 non-K	45 K / 4 non-K	$\chi^2(1) = 1.62$	0.203	V = 0.06
Year of study	220 1st–2nd / 238 3rd+	23 / 26	$\chi^2(1) = 0.02$	0.884	V = 0.01
College type	292 non-health / 162 health	37 / 12	$\chi^2(1) = 2.45$	0.118	V = 0.07
Perceived need for help	246 no / 212 yes	32 / 17	$\chi^2(1) = 2.40$	0.121	V = 0.07

Continuous variables were compared using Welch's t test. The mean difference in SSOSH total score was −2.09 (95% CI −3.93 to −0.25). The difference was confined to the reverse-worded component (mean difference −1.95, 95% CI −3.61 to −0.29); the positively worded component did not differ (mean difference −0.14, 95% CI −1.57 to 1.30). K = Kuwaiti. Perceived need for help is the participant's report of whether they felt they needed professional mental-health help in the preceding 12 months; it was collected in the parent survey and is used here only for this comparison and in the imputation model.

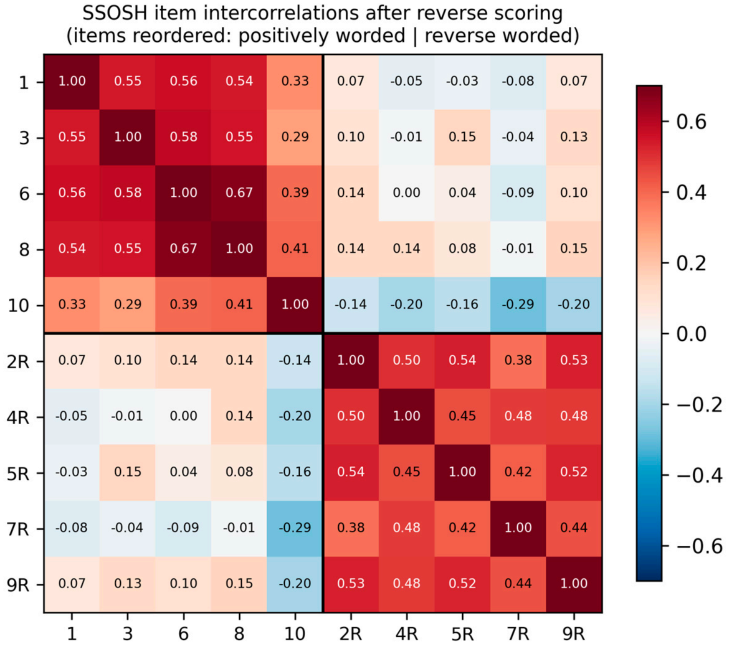

Figure S1. Intercorrelations among the ten scored SSOSH items (N = 507), with items reordered so that the five positively worded items appear first and the five reverse-worded items second. The heavy lines separate the two blocks. Within-block correlations are moderate to strong and positive; between-block correlations are close to zero or negative, which is the pattern that produces the two-factor solution reported in Section 3.2. Item 10 correlates positively with the other positively worded items and negatively with every reverse-worded item.

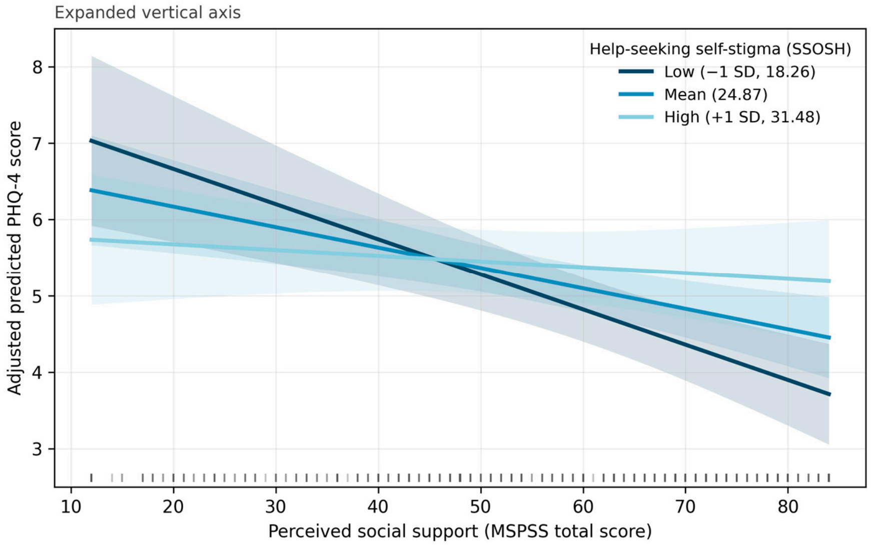

Figure S2. Adjusted PHQ-4 predictions by perceived social support and help-seeking self-stigma, expanded vertical axis. The figure presents the same model predictions and 95% confidence intervals as Figure 1 using an expanded vertical axis so that the convergence of the three conditional slopes is visible. Tick marks along the horizontal axis show observed MSPSS values.
